# Regulation of Zfp36 by ISGF3 and MK2 restricts the expression of inflammatory cytokines during necroptosis stimulation

**DOI:** 10.1038/s41419-024-06964-4

**Published:** 2024-08-08

**Authors:** Sahil Yadav, Rayan El Hamra, Norah A. Alturki, Ardeshir Ariana, Avni Bhan, Kate Hurley, Matthias Gaestel, Perry J. Blackshear, Alexandre Blais, Subash Sad

**Affiliations:** 1 Department of Biochemistry, Microbiology, and Immunology, Faculty of Medicine, University of Ottawa, Ottawa, ON Canada; 2 Clinical Laboratory Science Department, College of Applied Medical Sciences, King Saud University, Riyadh, Saudi Arabia; 3 Institute of Cell Biochemistry, Hannover Medical School, Hannover, Germany; 4 Signal Transduction Laboratory, National Institute of Environmental Health Sciences, Research Triangle Park, Durham, North Carolina United States of America; 5 Ottawa Institute of Systems Biology, Ottawa, ON Canada; 6 University of Ottawa, Centre for Infection Immunity and Inflammation, Ottawa, ON Canada; 7 University of Ottawa Brain and Mind Research Institute, Ottawa, ON Canada

**Keywords:** Interferons, Acute inflammation

## Abstract

Necrosome activation following TLR- or cytokine receptor-signaling results in cell death by necroptosis which is characterized by the rupture of cell membranes and the consequent release of intracellular contents to the extracellular milieu. While necroptosis exacerbates various inflammatory diseases, the mechanisms through which the inflammatory responses are regulated are not clear. We show that the necrosome activation of macrophages results in an upregulation of various pathways, including the mitogen-activated protein kinase (MAPK) cascade, which results in an elevation of the inflammatory response and consequent expression of several cytokines and chemokines. Programming for this upregulation of inflammatory response occurs during the early phase of necrosome activation and proceeds independently of cell death but depends on the activation of the receptor-interacting protein kinase-1 (RipK1). Interestingly, necrosome activation also results in an upregulation of IFNβ, which in turn exerts an inhibitory effect on the maintenance of inflammatory response through the repression of MAPK-signaling and an upregulation of *Zfp36*. Activation of the interferon-induced gene factor-3 (ISGF3) results in the expression of ZFP36 (TTP), which induces the post-transcriptional degradation of mRNAs of various inflammatory cytokines and chemokines through the recognition of AU-rich elements in their 3’UTR. Furthermore, ZFP-36 inhibits IFNβ-, but not TNFα- induced necroptosis. Overall, these results reveal the molecular mechanism through which IFNβ, a pro-inflammatory cytokine, induces the expression of ZFP-36, which in turn inhibits necroptosis and halts the maintenance of the inflammatory response.

## Introduction

Macrophages are a subset of myeloid cells that are present in various anatomical compartments of the body, where they play a frontline role in eliminating pathogens through the expression of high levels of inflammatory cytokines such as TNFα, IL-1, and IL-8 [1]. Inflammatory cytokines can also be induced by sterile triggers such as oxidized LDL [2]. While an acute cytokine storm is a beneficial host response against pathogens, chronic expression of inflammatory cytokines can lead to destruction of tissues, and consequent disease [3].

Various pathways of cell death have been implicated in inflammatory responses [4, 5]. The intrinsic and extrinsic pathways of apoptosis play fundamental roles during organismal development, and in the elimination of self-reactive immune cells [6]. The apoptotic blebs are rapidly cleared by neighboring macrophages through efferocytosis, and the process is not inflammatory [7]. In contrast to apoptosis, several pathways of inflammatory cell death have been revealed. Pyroptosis is an inflammatory pathway of cell death that is activated by inflammasome signaling [8]. Recognition of bacterial virulence factors by inflammasomes results in the activation of caspase-1/8/11, which results in the cleavage of pro-IL-1β and pro-IL-18 to active IL-1β and IL-18. This leads to the secretion of IL-1β and IL-18, and rupture of the cell membrane, which results in the release of intracellular damage-associated molecular patterns (DAMPS) such as mitochondrial DNA [9]. Necroptosis is another pathway of inflammatory cell death that is induced by TLR- and cytokine-receptor signaling in the context of inactive caspase-8 [10]. Necroptosis is induced following necrosome activation that is initiated by the phosphorylation of the receptor-interacting protein kinase-1 (RipK1), which results in the phosphorylation of other downstream interacting proteins such as RipK3 and mixed lineage kinase domain-like pseudokinase (MLKL) [11, 12]. Assembly of the necrosome leads to the trimerization of MLKL, which leads to the formation of MLKL-trimers, which relocate to the cell membrane and interact with PtdIns(4,5)P2, resulting in the leakage of cell membrane [13] and consequent release of intracellular DAMPs [14–17] and exacerbation of inflammation [18]. Accumulation of MLKL at the plasma membrane is a critical checkpoint in necroptosis [19].

While the inflammatory pathways of cell death play important roles in controlling infection, persistent activation of the necrosome exacerbates inflammatory bowel disease, liver injury, necrotizing pancreatitis, systemic inflammatory disorder, multiple sclerosis, atherosclerosis, necrotizing dermatitis, fatty liver disease and amyotrophic lateral sclerosis [20–27]. It is generally thought that the cell rupture following necroptosis and consequent spillage of the intracellular contents to the extracellular milieu leads to inflammation [18, 21]. In this report, we performed a transcriptomic analysis of macrophages during necrosome activation to reveal the molecular mechanisms that activate and regulate the inflammatory response that is induced following necrosome activation. We show that the necrosome activation induces the inflammatory response independently of cell death, and that this is regulated by type I interferon-induced expression of the zinc finger protein (ZFP)-36, also called tristetraprolin (TTP).

## Results

### Necroptosis stimulation induces an inflammatory storm

**Fig. 1:**
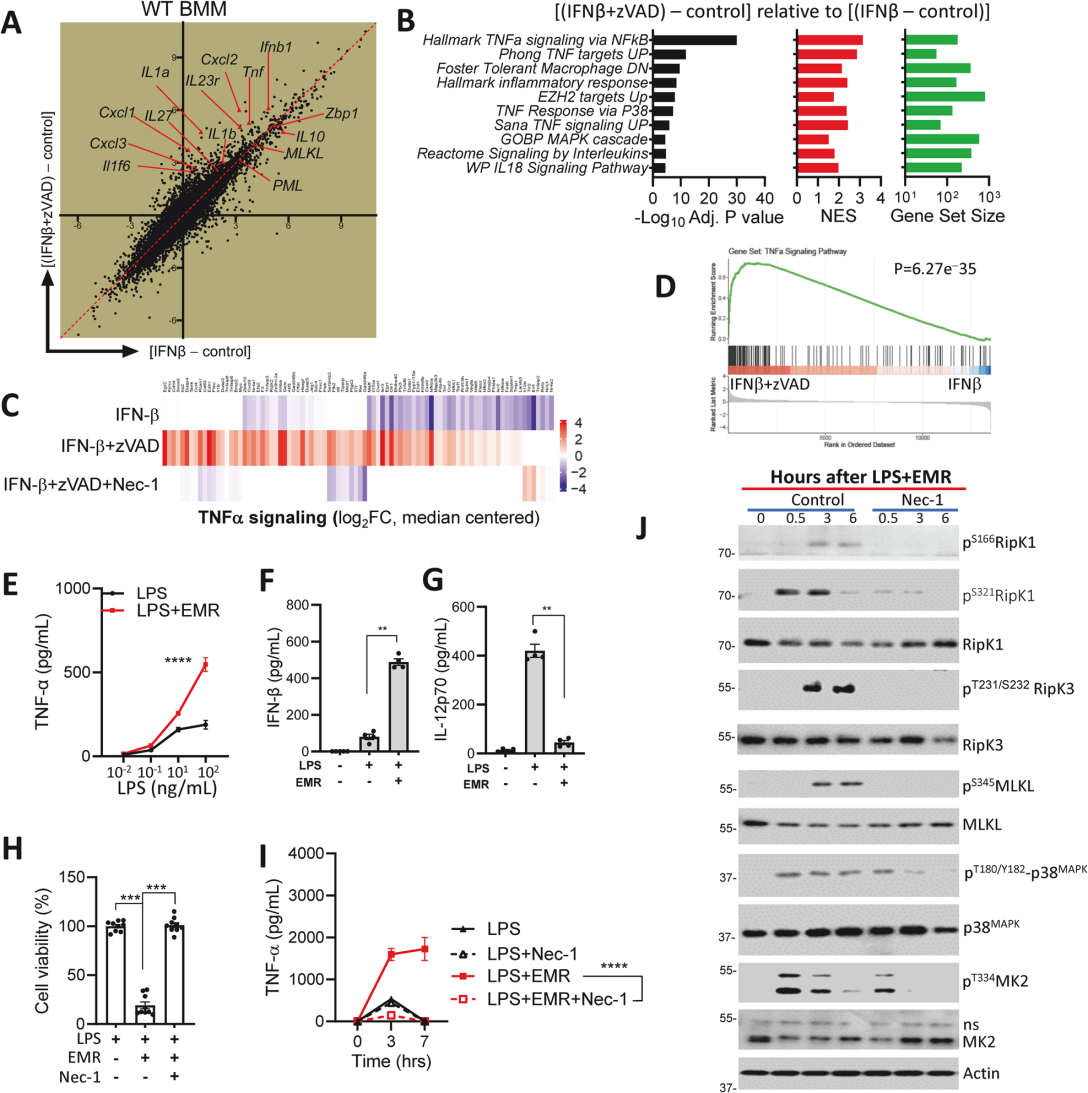
Necroptosis stimulation induces an inflammatory storm. WT BMDMs were treated with IFNβ (100 U/mL), zVAD-fmk (50 µM), and Nec-1 (10 µM). At 6 h, differential gene expression and GSEA were performed on microarray data comparing WT BMDMs treated with IFNβ, IFNβ + zVAD, and IFNβ + zVAD+Nec1 (**A**–**D**). Expression of TNFα was measured in the supernatants collected at 7 h after treating the WT BMDMs with EMR (10 µM) and different LPS concentrations (**E**). Expression of IFNβ, and IL-12p70 was measured in the supernatants collected at 7 h after treating the WT BMDMs with LPS (1 ng/mL) and EMR (10 µM) at 7 h (**F**, **G**). **H**, **I** WT BMDMs were stimulated with LPS (1 ng/mL), EMR (10 µM), Nec1-s (10 µM) and the impact on cell death was evaluated at 24 h by MTT (**H**). Secretion of TNFα was measured in supernatants collected at 7 h (**I**), and the activation of various proteins was evaluated by performing western blotting of cell extracts collected at various time intervals (**J**). Each experiment was repeated at least three times. (**P < 0.01, ***P < 0.001, ****P < 0.0001).

Necroptosis has been shown to result in the release of intracellular DAMPs, such as S100 proteins, mitochondrial DNA, and HMGB1, and the release of these into the extracellular milieu is speculated to result in the exacerbation of the inflammatory response that is often observed following necrotic cell death. We performed a transcriptomic analysis of bone marrow-derived macrophages (BMDMs) during necrosome stimulation at a time point (6 h) when cell death is not detectable (Fig. S1A). Differential gene expression analysis was performed on BMMs at 6 h following treatment with IFNβ, and IFNβ+zVAD. A 2D plot representing log_2_ fold-change of gene expression in cells treated with IFNβ relative to untreated WT BMDMs on the X-axis and log_2_FC of gene expression in cells treated with IFNβ+zVAD relative to untreated WT BMDMs on the Y-axis revealed a significant upregulation of various pro-inflammatory genes (e.g., *Tnfa, Il1, Ifnb, Cxcl1*) in cells stimulated with IFNβ+zVAD (Fig. 1A). In addition, gene set enrichment analysis (GSEA) revealed the upregulation of various pathways related to the inflammatory response (Figs. 1B–D and S1B–D). No significant modulation of pathways related to protein translation was detected. Analysis of the enriched leading edge gene sets revealed an upregulation of the TNFα signaling pathway during necroptosis stimulation, which resulted in an increased expression of various genes such as *TNFα, Cxcl1* and *Cxcl2*. Upregulation of the inflammatory response during necroptosis stimulation by IFNβ+zVAD treatment was significantly reduced in cells treated with Necrostatin-1 (Nec-1), which blocks the kinase activity of RipK1 and inhibits RipK1-RipK3 interaction [28] (Figs. 1C and S1B). Comparison of GSEA in cells treated with LPS+zVAD relative to LPS revealed similar upregulation of the inflammatory pathways (Fig. S1E).

Treatment of BMDMs with LPS and the pan-caspase inhibitor Q-VD-OPh or the caspase-8 inhibitor (zIETD-fmk) does not induce necroptosis [29]. Although zVAD-fmk is a pan-caspase inhibitor, it blocks the caspase-8–cFLIPs heterodimer more efficiently than the zIETD-fmk or Q-VD-OPh, hence favoring necroptosis [30, 31]. Recently, it has been reported that the caspase inhibitor Emricasan (EMR) promotes necroptosis at reduced concentrations, due to its increased specificity towards caspase-8, which results in better inhibition of the cFLIP-caspase-8 heterodimers [32]. We, therefore, treated cells with LPS and EMR and measured the impact on cytokine expression and cell death. Treatment of cells with LPS+zVAD or LPS + EMR resulted in similar secretion of TNFα in cell supernatants (at 6 h) (Fig. S1F) and cell death (24 h) (Fig. S1G). In correlation with increased transcription, the levels of secreted TNFα and IFNβ were increased in cells during necroptosis stimulation (treatment with LPS + EMR) (Fig. 1E, F). Necroptosis stimulation did not result in an upregulation of all cytokines, since there was no impact on the expression of the anti-inflammatory cytokine IL-10 (Fig. S1H). On the contrary, the expression of IL-12 (Fig. 1G) and IL-6 (Fig. S1I) was significantly suppressed during necroptosis stimulation. These results indicate that while there is a global augmentation of inflammatory response during necroptosis stimulation (Fig. 1D), the expression of some cytokines is selectively downregulated.

Induction of necroptosis by LPS (Fig. 1H), IFNβ (Fig. S1J), or TNFα (Fig. S1L) was inhibited by Nec-1. In contrast to IFNβ, treatment of cells with IFNγ did not induce necroptosis (Fig. S1K). Interestingly, treatment of cells with Nec-1 during necroptosis stimulation resulted in a significant reduction in the levels of secreted TNFα (Fig. 1I), indicating that the activation of RipK1 is responsible for the augmentation of inflammatory responses. Western blotting of cell extracts at various time intervals post-necroptosis stimulation revealed that Nec-1 inhibited the phosphorylation of RipK1 and its downstream targets RipK3 and MLKL as expected (Fig. 1J). Since the S321 phosphorylation of RipK1 has been reported to be mediated by the MAPK-activated protein kinase 2 (MAPKAPK2, or MK2) [33–35], and that MK2 promotes the stability of TNFα mRNA [36, 37], we tested the impact of Nec-1 on the activation (phosphorylation) of MK2. Our results indicate that Nec-1 also inhibited the activation of MK2 (Fig. 1J). In addition to the activation by p38MAPK [38], MK2 has also been shown to be activated by ERK1/2 [39, 40]. Our results indicate that the activation of ERK1/2 was also noticeably inhibited by Nec-1 during necrosome activation (Fig. S1M).

Overall, these results indicate that the RipK1-dependent p38^MAPK^ pathway promotes inflammation during necrosome activation.

### ISGF3 restricts TNFα expression during necroptosis stimulation

**Fig. 2:**
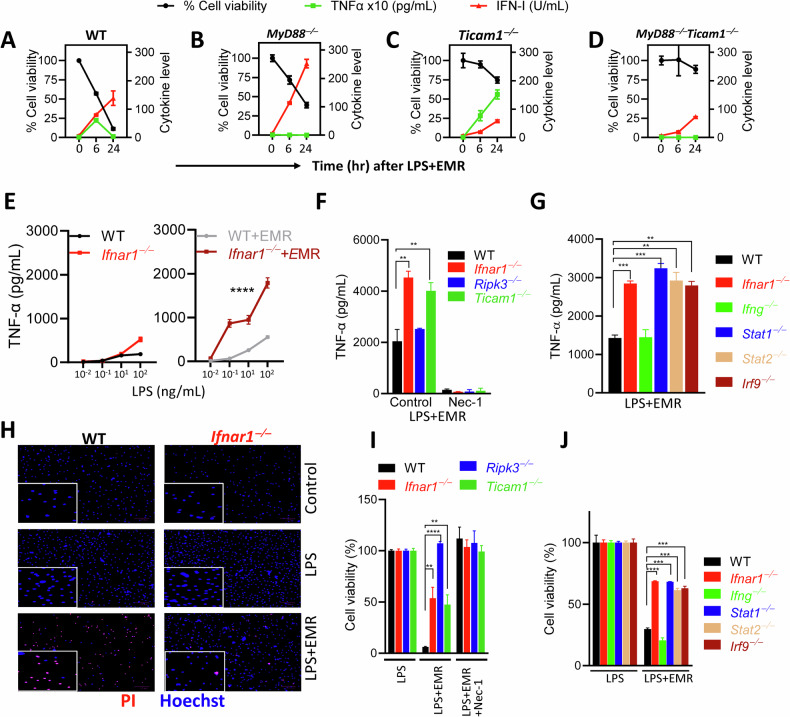
ISGF3 restricts TNFα expression during necroptosis stimulation. **A**–**D** WT, *MyD88*^*−/−*^, *Ticam1*^*−/−*^, and *Myd88*^*−/−*^*Ticam1*^*−/−*^ BMDMs were treated with LPS (1 ng/mL) and EMR (10 µM). At various time intervals cell death was measured by MTT, and expression of TNFα and IFN-1 was measured in the supernatants by ELISA and bioassay respectively. **E** TNFα levels were measured in the supernatants of WT and *Ifnar1*^*−/−*^ BMDMs at 7 h following treatment with different concentrations of LPS and EMR (10 µM). **F**, **G** TNFα levels were measured in the supernatants of BMDMs of various genotypes at 7 h following treatment with LPS (1 ng/mL), EMR (10 µM) and Nec-1 (10 µM). **H**–**J** Cell death was evaluated at 24 h by staining cells with PI and Hoechst (**H**) or by the MTT assay (**I**, **J**). Each experiment was repeated at least three times. (**P < 0.01, ***P < 0.001, ****P < 0.0001).

Type I interferon has been shown to be a key driver of the cytokine storm during viral infections [41], and we have previously reported that *Ifnar1*^*−/−*^ macrophages are significantly resistant to necroptosis [16]. We, therefore, evaluated the impact of the TLR-adapter proteins, MyD88 and TRIF, and the transcription factors downstream of IFNAR1 that might impact TNFα expression during necroptosis stimulation. TRIF-deficient (*Ticam1*^*−/−*^) BMDMs were highly resistant to necroptosis in response to TLR4 engagement, and this was bolstered further in macrophages deficient in both *MyD88* and *Ticam1* (Fig. 2A–D). In WT BMDMs, TNFα expression was detectable at 6 h, which was strongly reduced at 24 h (Fig. 2A). Since TNFα mRNA is highly susceptible to degradation [36, 42], it is possible that the reduction of TNFα levels at 24 h may be related to RNA decay. In *MyD88*^*−/−*^ macrophages, TNFα expression was undetectable, indicating that MyD88 is indispensable for the transcription of TNFα during necroptosis stimulation (Fig. 2B). On the other hand, *Ticam1*^*−/−*^ BMDMs displayed an augmentation in the expression of TNFα at 24 h post stimulation in comparison to WT or *MyD88*^*−/−*^ macrophages, implying that TRIF-signaling may promote the degradation of TNFα (Fig. 2C). The upregulation of TNFα in *Ticam1*^*−/−*^ BMDMs was abrogated by concomitant deletion of *MyD88* (Fig. 2D). In contrast to TNFα, the expression of type I interferon was promoted by TRIF signaling (Fig. 2A–D). These results indicate that the two key adapter proteins, MyD88 and TRIF, that promote TLR4 signaling have opposite impacts on TNFα expression. MyD88 promotes the early expression of TNFα, whereas TRIF possibly compromises the maintenance of TNFα.

In response to LPS stimulation, *Ifnar1*^*−/−*^BMDMs displayed only a minor increase in TNFα expression in comparison to WT cells (Fig. 2E). On the other hand, necroptotic stimulation (LPS + EMR) of *Ifnar1*^*−/−*^BMDMs resulted in a substantial increase in TNFα expression in comparison to WT cells (Fig. 2E). *Ifnar1*^*−/−*^ or *Ticam1*^*−/−*^macrophages displayed a similar augmentation in TNFα expression in contrast to WT cells, which was abrogated by treatment of cells with Nec-1 (Fig. 2F). Deficiency of the necrosome interacting protein RipK3 had no impact on TNFα expression (Fig. 2F). IFNAR1 signaling results in the phosphorylation driven assembly of STAT1, STAT2 and IRF9, collectively called ISGF3, which promotes the transcription of genes that harbor an ISRE element in the promoter [43, 44]. Our results indicate that *Stat1*^*−/−*^*, Stat2*^*−/−*^, or *Irf9*^*−/−*^macrophages express increased levels of TNFα in comparison to WT macrophages (Fig. 2G). Interestingly, IFN-γ, which induces cell signaling by promoting the binding of STAT1 to the promoters of genes that harbor a GAS element [43], did not impact the expression of TNFα (Fig. 2G). These results indicate that the IFNAR1-induced ISGF3 signaling is responsible for the reduction of TNFα levels during necroptosis stimulation. In contrast to the inhibitory impact of ISGF3 on TNFα expression, ISGF3 promoted the expression of IL-10 (Fig. S2A, B).

Staining of BMDMs with PI+Hoechst at 24 h post stimulation revealed that *Ifnar1*^*−/−*^macrophages are highly resistant to necroptosis (Fig. 2H). Very little cell death was detectable at 6 h post stimulation (Fig. S2C). *Ifnar1*^*−/−*^ or *Ticam1*^*−/−*^macrophages displayed a similar reduction in necroptosis following LPS + EMR stimulation in comparison to *RipK3*^*−/−*^ macrophages that displayed complete resistance (Fig. 2I). In addition, *Ifnar1*^*−/−*^, but not *Ticam1*^*−/−*^ macrophages displayed a significant reduction in necroptosis following stimulation by TNFα + EMR or IFNβ + EMR (Fig. S2D, E). Macrophages deficient in the ISGF3 members (*Stat1, Stat2*, or *Irf9*) displayed enhanced resistance to necroptosis induced by LPS + EMR (Fig. 2J) or TNFα + EMR (Fig. S2F). Overall, these results indicate that ISGF3 signaling promotes necroptosis of macrophages but restricts the expression of TNFα.

### Modulation of inflammatory response by IFNAR1- signaling during necroptosis stimulation

**Fig. 3:**
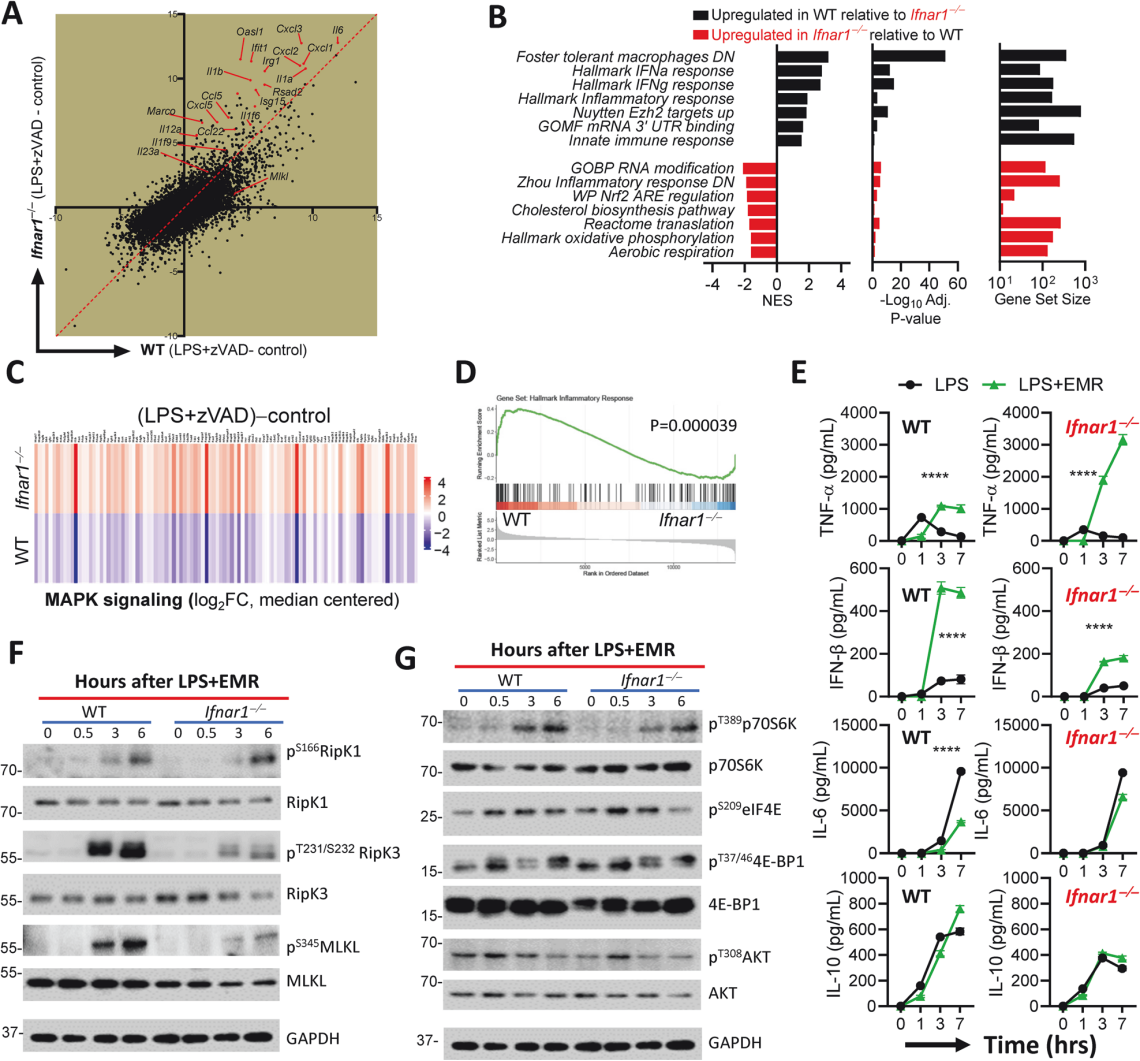
Modulation of the inflammatory response by Ifnar1 signaling during necroptosis stimulation. WT and *Ifnar1*^*−/−*^ BMDMs were treated with LPS (1 ng/mL) in the presence of zVAD-fmk (50 µM) for 6 h and differential gene expression and GSEA was performed on microarray data comparing WT BMDMs treated with LPS+zVAD with *Ifnar1*^*−/−*^ BMDMs treated with LPS+zVAD (**A**–**D**). **E** Expression levels of TNFα, IFNβ, IL-6, and IL-10 were measured in the supernatants collected after treating WT and *Ifnar1*^*−/−*^ BMDMs for 7 h with LPS (1 ng/mL) and EMR (10 µM). **F**, **G** Western blot analysis was performed in cell extracts collected from WT and *Ifnar1*^*−/−*^ BMDMs at different time intervals following treatment with LPS (1 ng/mL) and EMR (10 µM). Each experiment was repeated at least three times. (****P < 0.0001).

Microarray analysis revealed that the transcription of various inflammatory genes was upregulated during necrosome activation of *Ifnar1*^*−/−*^macrophages (Fig. 3A). As expected, the gene set enrichment analysis revealed a downregulation of type I and type II interferon response in *Ifnar1*^*−/−*^macrophages relative to WT cells (Fig. 3B). Interestingly, WT cells displayed a significant enrichment in the upregulation of the mRNA editing pathway relative to *Ifnar1*^*−/−*^cells. In contrast, the pathways related to ROS detoxification, RNA modification, cholesterol biosynthesis and protein translation were enriched in *Ifnar1*^*−/−*^macrophages relative to WT cells. A significant number of genes in the inflammatory and the MAPK pathways were up- or down-regulated by type I interferon signaling (Figs. 3C, D and S3A). We evaluated the impact of necroptosis stimulation on cytokine expression kinetically in WT versus *Ifnar1*^*−/−*^BMDMs. At 1 h post stimulation, the secretion of TNFα was reduced when cells were stimulated with LPS + EMR in comparison to LPS, and the situation was reversed at later time intervals (Fig. 3E). While the necroptosis stimulation of WT BMDMs with LPS + EMR resulted in increased expression of TNFα at later time intervals, this was augmented even further in *Ifnar1*^*−/−*^ BMDMs (Fig. 3E). The expression of IFNβ was also increased during necroptosis stimulation, but the levels were higher in WT cells, perhaps due to the lack of feed-forward IFNAR1-signaling in *Ifnar1*^*−/−*^ BMDMs. Similar to WT cells, the expression of IL-6 was slightly reduced in both WT and *Ifnar1*^*−/−*^ BMDMs (Fig. 3E).

Treatment of cells with Nec-1 at the time of LPS + EMR addition resulted in a reduction of TNFα levels in both WT and *Ifnar1*^*−/−*^macrophages (Fig. S3B). Interestingly, *Ifnar1*^*−/−*^BMDMs were resistant to Nec-1 when added at 1.5 h post LPS + EMR treatment in comparison to WT cells, which remained responsive to the inhibitory effect of Nec-1 (Fig. S3B). These results reveal significant differences in the role of RipK1 in promoting the kinetics of cytokine expression by WT versus *Ifnar1*^*−/−*^macrophages.

Western blotting of cell extracts demonstrated that the activation of RipK3 and MLKL was highly reduced in *Ifnar1*^*−/−*^ BMDMs, while the activation of RipK1 proceeded normally during necroptosis stimulation in *Ifnar1*^*−/−*^ BMDMs (Fig. 3F). In cells treated with LPS without EMR, there was no activation of RipK1, RipK3 or MLKL (Fig. S3C). Thus, the increased expression of TNFα by *Ifnar1*^*−/−*^ BMDMs does not appear to be mediated by the phosphorylation of RipK3 or MLKL. Various receptor ligand interactions result in the amplification of metabolism which can promote cytokine expression [45, 46]. This is initiated by the activation of the PI3K-AKT pathway, which promotes cell survival and proliferation through the activation of various downstream targets such as mTORC1. The ribosomal protein S6 kinase (S6K) and 4E-BP1 are the two direct targets of mTORC1, which are phosphorylated by mTORC1 [47]. 4E-BP1 inhibits protein translation by binding to the translation initiation factor eIF4E, and phosphorylation of 4E-BP1 by mTORC1 leads to the dissociation of 4E-BP1 from eIF4A and reinitiation of protein translation [48]. We performed western blot analysis of cells stimulated with LPS (Fig. S3D) or LPS + EMR (Fig. 3G) and did not detect any modulation of AKT, S6K, 4E-BP1, or eIF4E by IFNAR1-signaling. These results imply that the upregulation of TNFα expression in *Ifnar1*^*−/−*^ cells is not related to the modulation of translation or metabolism. One would also expect that an upregulation of metabolism might lead to an increased expression of numerous cytokines, which was not observed.

### Induction of Zfp36 (TTP) by Ifnar1 regulates TNFα expression and necroptosis

Looking for a mechanism that could explain the downregulation of transcripts of inflammatory genes by type I interferon signaling, we observed that the 3’UTR binding was a category which was enriched in the GSEA of WT cells relative to *Ifnar1*^*−/−*^ cells (Fig. 3B). *Zfp36* stood out as an interesting gene as it destabilizes mRNA transcripts that contain AU-rich elements (ARE) by facilitating their poly(A) tail removal, which results in the termination of protein expression [42]. Having observed that the expression of TNFα was amplified in *Ifnar1*^*−/−*^ macrophages at later time intervals of necroptosis stimulation (Fig. 3E), we considered the possibility that type I interferon may promote the degradation of transcripts that contain an AREs. The mRNA of various cytokines and chemokines such as *Tnfa, Il1b, Il6, Ifnb, Il10*, and *Cxcl1 harbor ARE in the 3’UTR*.

**Fig. 4:**
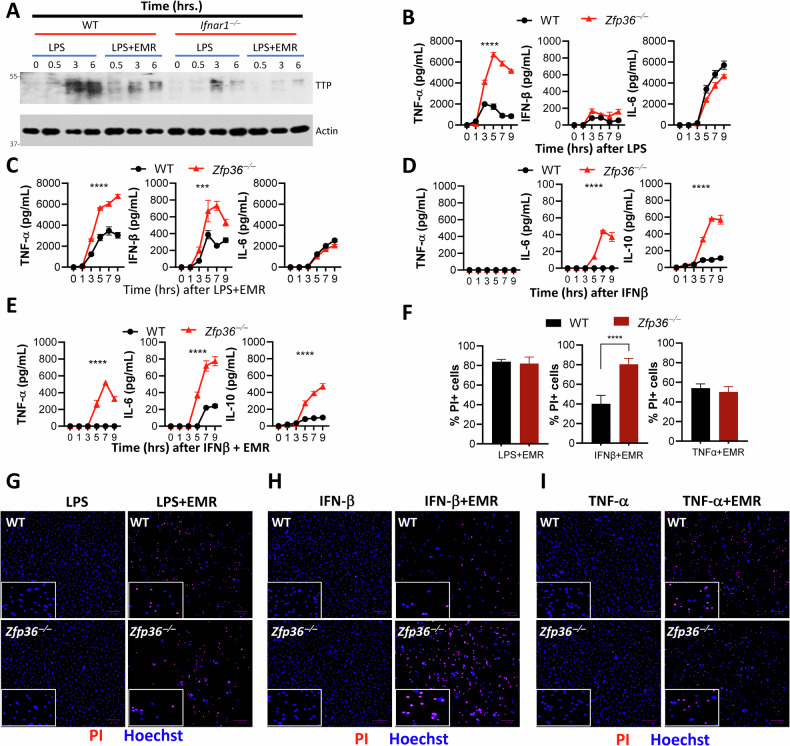
Induction of Zfp36 (TTP) by Ifnar1-signaling regulates TNFα expression and necroptosis. **A** Expression of TTP was analyzed by western blotting of cell extracts collected from WT and *Ifnar1*^*−/−*^ BMDMs at various time intervals following treatment with LPS (1 ng/mL) and/or EMR (10 µM). **B**–**E** Expression of TNFα, IFNβ, and IL-6 was measured in the supernatants collected at different time intervals after the treatment of WT and *Zfp36*^*−/−*^ BMDMs with LPS (1 ng/mL) (**B**), LPS (1 ng/mL)+EMR (10 µM) (**C**), IFNβ (10 ng/mL) (**D**), or IFNβ (10 ng/mL)+EMR (10 µM) (**E**). **F**, **G** Cell death was evaluated by staining of cells with Hoechst and PI at 24 h post treatment with LPS + /−EMR (**F**, **G**), IFNβ + /−EMR (**F, H**), TNFα ± EMR (**F**, **I**). Each experiment was repeated at least three times. (***P < 0.001, ****P < 0.0001).

We performed bioinformatics of the previously published ChiP-Seq dataset of macrophages treated with IFNβ from the NCBI SRA (GSE115435) [49], and observed that IFNβ-treatment induces the binding of ISGF3 members (STAT1, STAT2, and IRF9) to the ISRE site upstream of the *Zfp36* promoter (Fig. S4A). Our in vitro experiments indicated that the expression of *Zfp36* mRNA is reduced in *Ifnar1*^*−/−*^ cells substantially during necroptosis stimulation (Fig. S4B, C). The expression of TTP, the gene product of *Zfp36*, was reduced during necroptosis stimulation of WT macrophages, and was reduced even further in *Ifnar1*^*−/−*^ macrophages as detected by western blotting (Fig. 4A). *Zfp36*^*−/−*^ BMDMs expressed increased levels of TNFα following stimulation by LPS (Fig. 4B) or LPS + EMR (Fig. 4C). The expression of IFNβ was highly upregulated only during stimulation with LPS + EMR, and this was upregulated in *Zfp36*^*−/−*^ macrophages (Fig. 4C). Stimulation with IFNβ failed to induce TNFα expression in WT or *Zfp36*^*−/−*^ macrophages (Fig. 4D). On the other hand, TNFα expression was induced by IFNβ only during necroptosis stimulation (i.e., treatment with IFNβ + EMR) of *Zfp36*^*−/−*^, but not in WT macrophages (Figs. 4E and S4D). Treatment of cells with Nec-1 abrogated the induction of TNFa expression in *Zfp36*^*−/−*^ cells (Fig. S4D). Expression of IL-6 and IL-10 was also upregulated in *Zfp36*^*−/−*^ macrophages stimulated with IFNβ regardless of co-treatment with EMR (Fig. S4D). Interestingly, *Zfp36*^*−/−*^ BMDMs underwent increased necroptosis only when stimulated with IFNβ + EMR (Figs. 4F–I and S4E).

**Fig. 5:**
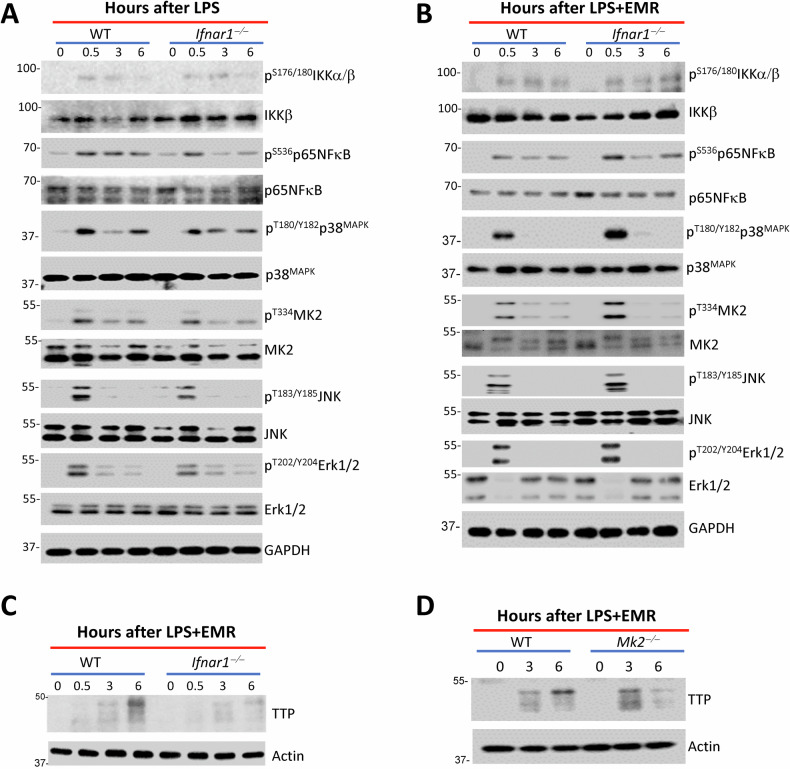
Modulation of MK2 and TTP by Ifnar1-signaling during necroptosis stimulation. WT and *Ifnar1*^*−/−*^ BMDMs (**A**–**C**) were stimulated with LPS (1 ng/mL) (**A**) or LPS (1 ng/mL)+EMR (10 µM) (**B**, **C**). Cell extracts were collected at various time intervals and the activation of signaling proteins was evaluated by western blot analysis. **D** WT and *Mk2*^*−/−*^ BMDMs were stimulated with LPS (1 ng/mL)+EMR (10 µM). Cell extracts were collected at various time intervals and the expression of TTP was evaluated by western blot analysis. Each experiment was repeated at least three times.

Western blotting of cell extracts did not reveal any modulation in the activation of NFκB or MAPK pathways by type I interferon signaling following LPS-treatment (Fig. 5A). On the other hand, there was an increase in the activation of the MAPK pathway during necroptosis stimulation of *Ifnar1*^*−/−*^ macrophages (Figs. 5B and S5). The induction in the expression of TTP was compromised in *Ifnar1*^*−/−*^ macrophages (Fig. 5C). MK2 has been shown to phosphorylate TTP, increase its stability, but prevent its mRNA binding [50]. We observed that the expression of TTP was reduced at 6 h following necrosome activation of *Mk2*^*−/−*^ macrophages (Fig. 5D). Taken together, these results indicate that TTP plays an important role in regulating the expression of TNFα and cell death by necroptosis mainly during necroptosis stimulation by type I interferon.

### Abrogation of MK2 restricts TNFα expression and augments necroptosis in *Ifnar1*^*−/−*^ macrophages

**Fig. 6:**
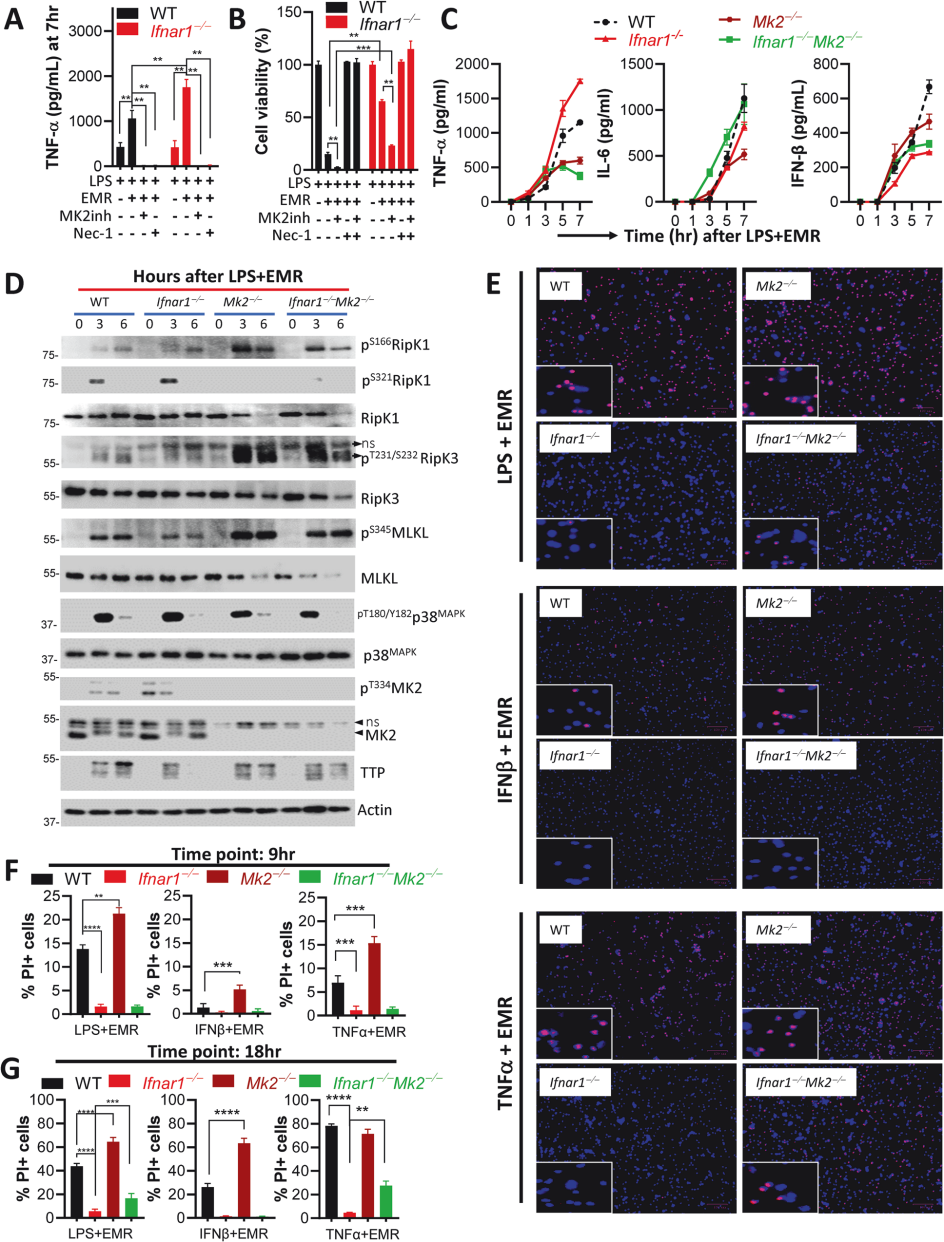
Abrogation of MK2 restricts TNFα expression and augments necroptosis in *Ifnar1*^*−/−*^ macrophages. **A**, **B** WT and *Ifnar1*^*−/−*^ BMDMs were stimulated with LPS (1 ng/mL), EMR (10 µM) and MK2 inhibitor (III, 5 µM), and the impact on TNFα secretion (**A**) and cell viability (**B**) was evaluated at 7 h and 24 h respectively. **C**–**G** WT, *Ifnar1*^*−/−*^, *Mk2*^*−/−*^, and *Ifnar1*^*−/−*^*Mk2*^*−/−*^ BMDMs were stimulated with LPS (1 ng/mL)+EMR (10 µM). Cytokine expression was evaluated at various time intervals by ELISA (**C**). Activation of signaling proteins was evaluated by western blot analysis of cell extracts collected at various time intervals (**D**). Cell death was evaluated by staining cells with Hoechst and PI following stimulation of WT, *Ifnar1*^*−/−*^, *Mk2*^*−/−*^, and *Ifnar1*^*−/−*^*Mk2*^*−/−*^ BMDMs with LPS (1 ng/mL)+EMR (10 µM), IFNβ (10 ng/mL)+EMR (10 µM), and TNFα (50 ng/mL)+EMR (10 µM) at 9 h (**F**) or 18 h (**E**, **G**). Each experiment was repeated at least three times. (*P < 0.05, **P < 0.01, ***P < 0.001, ****P < 0.0001).

Since MK2 plays a critical role in promoting the maintenance of TNFα expression [36, 37], and since we observed that the activation of MK2 is modulated during the necroptosis stimulation of *Ifnar1*^*−/−*^ macrophages, we therefore inhibited the p38^MAPK^ pathway and evaluated its impact on TNFα expression and necroptosis. Inhibition of MK2 by inhibitor III blocked TNFα expression (Fig. 6A) but enhanced necroptosis (Fig. 6B) of both WT and *Ifnar1*^*−/−*^ macrophages. Similar results were obtained with the p38^MAPK^ inhibitor (LY2228820) (Fig. S6A, B). Necroptosis of cells was rescuable by co-treatment of cells with the RipK3 inhibitor (GSK872) (Fig. S6C). To probe this pathway further we generated mice that are deficient in both *Ifnar1* and *Mapkapk2* (*Mk2*). Concomitant deficiency of *Mk2* abrogated the elevation of TNFα expression during the necrosome signaling of *Ifnar1*^*−/−*^ macrophages (Fig. 6C). In *Mk2*^*−/−*^ macrophages, the activating phosphorylation (S166) of RipK1 was upregulated, and this was reduced in *Ifnar1*^*−/−*^*Mk2*^*−/−*^ macrophages (Fig. 6D). This suggests that the upregulation in S166 phosphorylation of RipK1 in *Mk2*^*−/−*^ macrophages is partly dependent on IFNAR1 signaling. The inhibitory (S321) phosphorylation of RipK1, which was slightly increased in *Ifnar1*^*−/−*^ macrophages, was abrogated in *Mk2*^*−/−*^ or *Ifnar1*^*−/−*^*Mk2*^*−/−*^ macrophages (Fig. 6D). The downstream phosphorylation of RipK3 and MLKL was upregulated in *Mk2*^*−/−*^ macrophages, which was slightly reduced in *Ifnar1*^*−/−*^*Mk2*^*−/−*^ macrophages (Fig. 6D).

MK2 exerted an inhibitory impact on necroptosis during activation by LPS or IFNβ, (Fig. 6E–G). The inhibitory impact of MK2 during necroptosis stimulation by TNFα was detectable only at 9 h (Figs. 6F and S6D) but not at 18 h post stimulation (Fig. 6E, G). The deficiency of *Mk2* in *Ifnar1*^*−/−*^ macrophages resulted in increased necroptosis induced by LPS or TNFα (Fig. 6E–G).

Taken together, these results indicate that the modulation of MK2 and TTP by IFNAR1 signaling regulates the inflammatory cytokine milieu during necrosome activation.

### IFNAR1 signaling promotes the degradation of TNFα mRNA

**Fig. 7:**
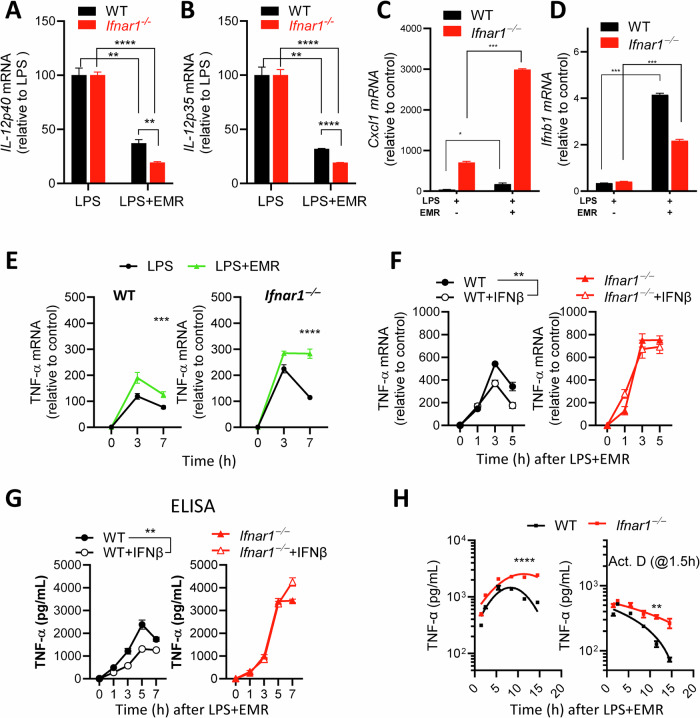
Ifnar1-signaling promotes the degradation of TNFα mRNA. **A**–**D** Expression of *Il-12*, *Cxcl11*, and *Ifnb1* mRNA were measured by qRT-PCR analysis at 3 h following treatment of WT and *Ifnar1*^*−/−*^ BMDMs with LPS (1 ng/mL) and EMR (10 µM). **E**, **F** Expression of *Tnfa* mRNA was measured by qRT-PCR analysis at various time intervals post treatment of WT and *Ifnar1*^*−/−*^ BMDMs with LPS (1 ng/mL), EMR (10 µM), and IFNβ (10 ng/mL). **G**, **H** Expression of TNFα was measured by ELISA in the supernatants collected at different time intervals following treatment of WT and *Ifnar1*^*−/−*^ BMDMs with LPS (1 ng/mL), EMR (10 µM), and IFNβ (10 ng/mL), or Actinomycin D (2 µM). Each experiment was at least repeated three times. (*P < 0.05, **P < 0.01, ***P < 0.001, ****P < 0.0001).

We performed qRT-PCR analysis to measure the transcript levels of some of the genes that were upregulated (*Cxcl1, Ifnb1, Tnfa*) or downregulated (*IL-12*) during necroptosis stimulation. The mRNA level of IL-12 was reduced during necroptosis stimulation, and the reduction was greater in *Ifnar1*^*−/−*^ macrophages (Fig. 7A, B). In contrast, the transcript level of *Cxcl1* was upregulated during necrosome activation, and *Ifnar1*^*−/−*^ macrophages displayed more upregulation than WT cells (Fig. 7C). Expression of *Ifnb1* transcript was also enhanced during necroptosis stimulation, but in this case the WT cells displayed greater upregulation relative to *Ifnar1*^*−/−*^ cells (Fig. 7D). This could be due to the lack of feedforward signaling by IFNβ in *Ifnar1*^*−/−*^ macrophages. *Tnfa* mRNA was enhanced during necrosome activation, and this was increased further in *Ifnar1*^*−/−*^ macrophages (Fig. 7E). Treatment with IFNβ reduced the transcription (Fig. 7F) and translation (Fig. 7G) of *Tnfa* in WT but not in *Ifnar1*^*−/−*^ macrophages.

**Fig. 8:**
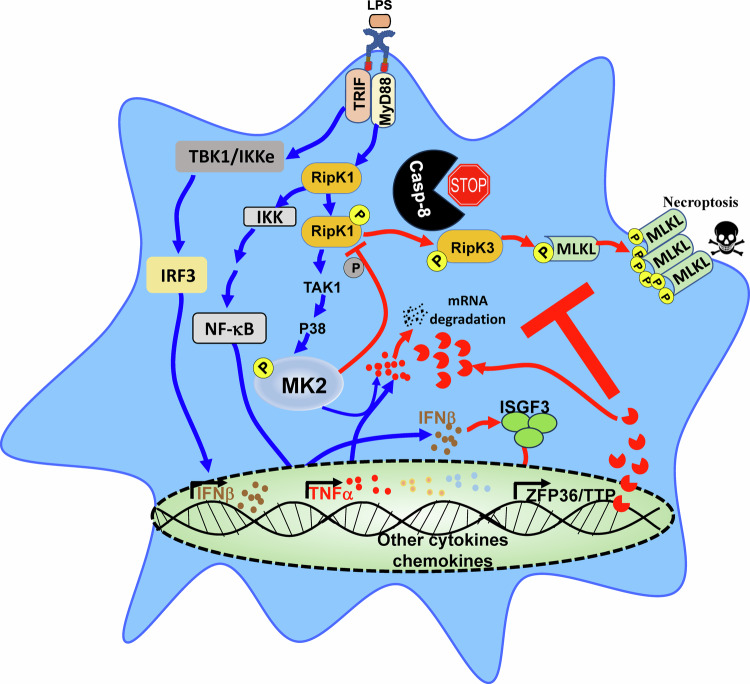
Ifnar1 signaling modulates inflammation during necrosome activation. Necrosome signaling results in the phosphorylation of RipK1 which promotes the activation of the MAPK pathway independently of cell death leading to increased inflammatory response. Expression of type I interferon through TRIF-signaling results in the activation of the ISGF3 transcriptional complex which induces the expression of TTP which causes the degradation of mRNA of various inflammatory cytokines and chemokines. Thus, IFNAR1-signaling promotes cell death by necroptosis but limits the expression of excessive inflammation.

We treated cells with Actinomycin D at 1.5 h post necrosome activation to inhibit gene transcription and evaluated the impact on the levels of *Tnfa* transcripts at various time intervals subsequently. Inhibition of transcription revealed that *Tnfa* transcripts underwent reduced degradation in *Ifnar1*^*−/−*^ macrophages (Fig. 7H). Taken together, these results indicate that necroptosis stimulation promotes RipK1-dependent activation of the p38^MAPK^ pathway, which promotes inflammatory response independently of cell death. This upregulation of inflammation is partly suppressed by IFNAR1-signaling through ISGF3, which promotes the transcription of the RNA-destabilizing protein TTP (Fig. 8).

## Discussion

Necroptosis of cells has been shown to exacerbate various acute and chronic inflammatory diseases [20–27], and it been suggested that this is perpetuated by the spillage of intracellular contents to the extracellular milieu, which engages additional signaling pathways that promote inflammation [51]. In this report we demonstrate that necroptosis signaling results in an augmentation of the inflammatory response independently and prior to the commencement of cell death. We also elucidate a regulatory mechanism driven by ISGF3-MK2-dependent TTP expression, which fine-tunes the inflammatory response during necroptosis signaling through a post-transcriptional mechanism.

While the premature cell death of macrophages can impact the expression of cytokines [52], the augmentation in the expression of inflammatory cytokines during necroptosis signaling that we have shown here is not related to cell death for the following reasons: (1) increase in cytokine expression occurred prior to the commencement of cell death, (2) cytokine expression was augmented in *RipK3*^*−/−*^ cells that were completely resistant to cell death, (3) *Ifnar1*^*−/−*^ or *Ticam1*^*−/−*^ cells displayed reduced cell death but the cytokine expression was still augmented, and (4) Increased expression of inflammatory cytokines was also observed in *Mlkl*^*−/−*^ mice that are resistant to necroptosis [53].

It was previously reported that the activation of necroptosis signaling promotes the expression of several inflammatory cytokines and chemokines independently of cell death, which was dependent on the kinase activity of RipK1 and RipK3 [53]. While we also observed that necroptosis signaling the expression of TNFα was dependent on the kinase activity of RipK1, we failed to detect any involvement of RipK3 in the augmentation of TNFα production. In our experiments, the expression of TNFα was increased in both WT and *RipK3*^*−/−*^ cells to the same degree. Interestingly, the elevation of TNFα expression during necroptosis signaling was not dependent on MLKL [53], implying that the complete necrosome platform is not required for the augmentation of inflammation. Since the necrosome formation is compromised in the absence of RipK3, this indicates that the translocation and phosphorylation of RipK1 towards the necrosome pathway may be responsible for upregulation of inflammation. In the absence of necroptosis signaling, RipK1 has been shown to promote cell survival and inflammation [54–59]. Addition of the K63 ubiquitin chains on RipK1 results in the recognition of the ubiquitin chains by TAB2, which in turn leads to the recruitment and activation of TAK1 and IκB. This leads to the degradation of IκB which in turn promotes the activation of NFκB [59, 60]. Although the specific pathway that promotes the activation of the MAPK pathway via RipK1 is not that clear, it has suggested that the activation of TAK1 promotes the phosphorylation and activation of MAPK [59, 60]. It has also been suggested that RipK1 may mediate the p38^MAPK^ activation by recruiting the MEKK3 [60].

It is well established that the MyD88 adapter protein that operates downstream of TLR4 is indispensable for the expression of TNFα, whereas the TRIF adapter is required for the expression of type I interferon [61]. Our results uphold this paradigm during stimulation of cells with LPS or LPS + EMR. Surprisingly, it was reported that the upregulation of TNFα during TLR4- or TLR4-induced necroptosis signaling was abrogated in *Ticam1*^*−/−*^ cells [53]. Considering that MyD88 is indispensable for the TLR4-induced expression of TNFα, it is unclear how this role can be taken over by TRIF during necroptosis signaling. In contrast, a later study reported that the activation of necroptosis signaling results in increased expression of TNFα in cells with compromised TRIF signaling [62], indicative of an inhibitory role of TRIF in the elevation of TNFα expression. It has also been reported that the kinase activities of RipK1 and RipK3 promote the production of IFNβ during necroptosis signaling through the IRF/TBK1 axis [63]. We observed that the expression of TNFα during the necroptosis signaling of *Ifnar1*^*−/−*^ and *Ticam1*^*−/−*^ cells was elevated to the same degree, indicating that TRIF signaling inhibits rather than augments TNFα expression.

Since TNFα signaling is an important mediator of inflammatory responses that influences various disease outcomes [64], we evaluated how the expression of TNFα is modulated during necroptosis signaling. While our data indicates that the necroptosis signaling results in the upregulation of various genes related to the TNFα signaling and the MAPK pathway, we also observed that the necroptosis signaling had a negative impact on the expression of several other cytokines such as IL-12 and IL-6. In fact, the expression of IL-12 was drastically inhibited during necroptosis signaling. Further studies will be needed to determine whether the reduced expression of IL-12 is a biomarker for necrosome activation in vivo. It was reported that the expression of the anti-inflammatory cytokine IL-10 was upregulated during the necroptosis signaling of *Ifnb*^*−/−*^ cells [62]. In contrast, we observed that the expression of IL-10 was downregulated in *Ifnar1*^*−/−*^ cells that were stimulated with LPS or LPS + EMR. It is well established that type I interferon promotes the expression of IL-10 [65]. We analyzed the published ChiP-Seq data of BMMs stimulated with IFNβ [49] and observed that there are multiple ISRE sites in the promoter region of IL-10, which explains the reduced expression of IL-10 by *Ifnar1*^*−/−*^ cells. Thus, the impact of necroptosis signaling on the upregulation of cytokines does not appear to be global, and additional mechanisms exist that modulate the expression of various cytokines separately. We have shown here that ISGF3 signaling induces the expression of TTP, which promotes the degradation of TNFα. It has been previously reported that TTP promotes the degradation of various cytokines/chemokine mRNAs that harbor the AU-rich elements (AREs) at the 3-UTR (e.g., *Tnfa, Il1a, Il1b, Il6, Ifnb, Il10, Cxcl1, Ccl4, Ccl2, Ccl3*) [42].

We have previously reported that during the necrosome signaling of macrophages the phosphorylation of RipK1 is similar between WT versus *Ifnar1*^*−/−*^ cells [16], whereas the phosphorylation (activation) of RipK3 and MLKL is compromised in *Ifnar1*^*−/−*^ cells, which results in increased resistance of *Ifnar1*^*−/−*^ cells against necroptosis [16]. Since the phosphorylation of RipK1 is similar in WT and *Ifnar1*^*−/−*^ cells, and the inflammatory response is elevated during the necroptosis signaling of *Ifnar1*^*−/−*^ cells, this suggests that the kinase activity of RipK1 may not be responsible for the augmentation of TNFα expression in *Ifnar1*^*−/−*^ cells. Indeed, we observed that *Ifnar1*^*−/−*^ cells were refractory to the inhibitory effects of Nec-1 when added at 1.5 h post-stimulation.

Also, it has been reported that necroptosis involves the phosphorylation of RipK3 [11], leading to subsequent phosphorylation and oligomerization of MLKL [13, 66, 67]. This activated MLKL then translocates to the plasma membrane, ultimately resulting in cell membrane rupture, an indispensable characteristic of necroptosis [13]. The unexpected finding observed in our experiments is that the treatment of *Ifnar1*^*−/−*^*Mk2*^*−/−*^ cells with LPS + EMR results in significant phosphorylation of RipK3 and MLKL, yet the level of necroptosis is still lower in comparison to WT cells under the same conditions (Fig. 6D–F), underscoring the complexity of the molecular pathways governing necroptotic cell death [68]. These interesting results encourage a deeper investigation into the nuanced roles of the intricate signaling network involved in necroptosis. As previously highlighted, the precise subcellular localization of phospho-MLKL emerges as a pivotal determinant in unraveling the regulatory mechanisms of necroptosis [19]. Our data suggest that despite robust phosphorylation events, the subcellular distribution of phospho-MLKL in *Ifnar1*^*−/−*^*Mk2*^*−/−*^ cells may deviate from the expected pattern, resulting in the observed attenuation of necroptotic outcomes. However, a comprehensive understanding of this phenomenon requires further experimental exploration, including detailed subcellular fractionation studies and advanced imaging techniques. Clarifying the spatial dynamics of phospho-MLKL within the cellular milieu during necroptosis is imperative for refining our comprehension of the underlying mechanisms and may offer new insights into potential regulatory checkpoints in this intricate cell death pathway.

Our results indicate that the transcription and translation of TNFα is upregulated during necrosome activation of macrophages. There have been contradictory reports regarding the translation of TNFα during the necrosome activation of WT macrophages, with one report indicating that the TNFα protein levels are upregulated [53], whereas the other report showed that the translation of *Tnfa* mRNA is not upregulated [62]. The later report indicated that the IFNAR1-signaling in WT cells, downstream of TRIF, is responsible for the suppression of the translation of *Tnfa* mRNA. The reasons for these contradictory results in WT cells are not clear but could be related to differences in basal type I interferon levels, which might exert their impact through tonic IFNAR1 signaling to suppress the protein levels. We have observed that the *Tnfa* mRNA and protein levels are upregulated in *Ifnar1*^*−/−*^ cells; however, our results indicate that this is due to a post-transcriptional and pre-translational mechanism that involves the expression of the mRNA degrading protein TTP (*Zfp36*) downstream of ISGF3.

GSEA of *Ifnar1*^*−/−*^ cells revealed a major downregulation of the innate immune response including the type I and type II interferon responses, which is expected since type I interferon is a major mediator of innate immune response [41]. On the other hand, there was an upregulation of various pathways such as RNA modification, antioxidant defense, and mitochondrial metabolism in *Ifnar1*^*−/−*^ cells, which can influence the expression of cytokines. A prior report indicated that the upregulation of cytokines during the necrosome activation of *Ifnar1*^*−/−*^ cells is related to increased protein translation [62]. Necroptosis stimulation was shown to result in increased puromycin incorporation by cells. However, the uptake of puromycin was similar in WT versus *Ifnβ*^*−/−*^ cells [62]. While the GSEA of our data indicated the enrichment of the mitochondrial translation in *Ifnar1*^*−/−*^ cells, we did not observe any enrichment in the eIF2 pathway and there was no detectable increase in the activators (p70S6K, eIF4E, AKT) or inhibitor (4E-BP1) of protein translation in *Ifnar1*^*−/−*^ cells during necrosome activation. Furthermore, if increased protein translation was responsible for the elevated inflammatory response in *Ifnar1*^*−/−*^ cells, then one would expect a global upregulation of protein expression, which is not the case. Type I interferon signaling has been shown to promote, rather than inhibit, the core metabolism in myeloid cells [46].

Our results indicate that IFNAR1 signaling impacts cytokine expression during necroptosis stimulation through the modulation of the p38^MAPK^-TTP axis. We have shown that ISGF3 signaling promotes the expression of TTP, which is an RNA-binding protein that controls the stability of mRNA of various transcripts that contain an ARE in the 3’ UTR [42]. Downstream of p38^MAPK^ activation, MK2 and MK3 phosphorylate TTP at various sites, leading to increased stability but compromised activity of TTP [69]. This phosphorylation negatively affects the ability of TTP regulate TNFα, which results in an increase in TNFα levels [69, 70]. When the p38^MAPK^ is inhibited, the phosphorylation and stability of TTP is compromised, which results in poor TNFα levels. We have previously reported that TTP inhibits necroptosis only when cells are stimulated with LPS+zVAD+p38^MAPK^ inhibitor [29]. We now show that TTP inhibits necroptosis only when cells are stimulated with IFNβ + EMR. This suggests a context-dependent regulation of necroptosis by TTP. In addition to modulating the activity and stability of TTP, MK2 mediates the inhibitory phosphorylation (S321) and inhibits the necrosome activating phosphorylation (S166) of RipK1, which is in agreement with previous studies [33–35]. Interestingly, the concomitant deficiency of *Ifnar1* in *Mk2*^*−/−*^ cells results in a reduction in the necrosome activating S166 phosphorylation of RipK1, without having any impact on the inhibitory phosphorylation, thereby uncoupling the inhibitory and stimulatory phosphorylation of RipK1 during necrosome activation.

Interferons have bene previously reported to promote the induction of TTP to limit the expression of inflammatory cytokines [71]. A gamma-activating sequence (GAS) was reported in the promoter of *Zfp36* [71]. However, this induction of TTP required the concomitant addition of the p38^MAPK^ agonist anisomycin. While we observed that the addition of the caspase inhibitor EMR results in the activation of the p38^MAPK^ pathway in cells treated with IFNβ or LPS, the induction of TTP was reduced during necroptosis activation of WT cells and reduced even further during necroptosis activation of *Ifnar1*-deficient cells. We did not detect any role of IFN-γ in inducing necroptosis and show that the upregulation of TNFα during necroptosis stimulation is compromised in cells that are deficient in type I interferon signaling (e.g., *Ifnar1*^*−/−*^*, Stat1*^*−/−*^*, Stat2*^*−/−*^, and *Irf9*^*−/−*^) but not in type II interferon signaling (*Ifng*^*−/−*^). In support of this we have revealed an ISRE in the promoter of *Zfp36*. It was reported that IL-10 promotes TTP induction through the activation of STAT3 which impacted the expression of IL-6, IL-12, and IL-23, but not TNFa [72]. In our work necroptosis stimulation of cells resulted in increased expression of TNFα but not IL-6 or IL-12, indicating that the IL-10/STAT3 axis may not be responsible for the upregulation of inflammatory response during necroptosis stimulation.

We have revealed the dichotomous role of type I interferon signaling in necroptosis. While IFNβ induces the transcription of MLKL to promote necroptosis [73], it also induces the expression of TTP to inhibit necroptosis. Interestingly, IFNβ signaling has been previously reported to inhibit inflammasome signaling [74], and a later study reported that TTP inhibits NLRP3-dependent inflammasome signaling [75].

We have highlighted the complex relationship between TNFα and IFNβ signaling in the regulation of inflammatory response during necroptosis signaling. While necroptosis signaling by multiple TLR’s is dependent on TNFα- [76] and- Ifnar1- [16] signaling, the molecular relationship between these two pathways in necroptosis induction in response to the same TLR engagement is not clear. Necroptosis of macrophages following TLR4 was shown to be dependent on TNF-R2 [77], and we have also confirmed this to be true. However, we failed to detect any modulation of TNF-R2 by IFNβ signaling. While IFNβ induces necroptosis, it also inhibits the expression TNFα, yet the necroptosis induction is dependent on both Ifnar1 as well as TNF-R2. We have shown here that *Ifnar1*-deficient macrophages remain resistant to necroptosis despite expressing higher levels of TNFα relative to WT cells. It is likely that a key downstream signaling pathway of necroptosis is blocked in *Ifnar1*-deficient cells. Previously it was reported that the reduced levels of MLKL in *Ifnar1*-deficient cells may lead to resistance to necroptosis [73]; however, increasing the level of MLKL in *Ifnar1*-deficient cells does not seem to rescue the phenotype [77].

In conclusion, we have shown that the commencement of the necroptosis program results in the activation of RipK1, which leads to the upregulation of MAPK pathway that promotes increased expression of various inflammatory cytokines independently of cell death. This upregulation of inflammatory response is counterbalanced by the expression of type I interferon during necrosome activation. The crosstalk between IFNAR1 signaling, the MAPK pathway, and post-transcriptional regulation through ZFP36 plays a crucial role in fine-tuning the balance between inflammatory responses and cell death. While type I interferon induces necroptosis, it also induces the expression of TTP, which causes the post-transcriptional degradation of various important inflammatory cytokines such as TNFα. In the absence of this preventative mechanism, necrosome signaling may lead to even greater toxicity to the host.

### Experimental procedures

#### Ethics approval and consent to participate

Experiments were performed in accordance with the Canadian Council on Animal Care (CCAC) guidelines and approved by the University of Ottawa Animal Care Committee (BMI#3590).

#### Mice

C57BL6/J (Jax #000664), *Ifnar1*^*−/−*^ (Jax #020288), *Ticam1*^*−/−*^ (Jax #005037), *MyD88*^*−/−*^ (Jax #009088), *Stat1*^*−/−*^ (Jax #012606), *Stat2*^*−/−*^ (Jax #023309) were obtained from Jackson Labs (Bar Harbor, USA). *Mk2*^*−/−*^ mice were obtained from Dr. Matthias Gaestel [36] (Hannover Medical School). *Zfp36*^*−/−*^ mice were obtained from Dr. Perry Blackshear [78] (National Institute of Environmental Sciences, NIH, USA). *RipK3*^*−/−*^ were a kind gift of Dr. Vishva Dixit (Genentech, San Francisco, CA, USA). *Irf9*^*−/−*^ mice were obtained from Dr. Karen Mossman (McMaster University). *MyD88*^*−/−*^*Ticam1*^*−/−*^ and *Ifnar1*^*−/−*^*Mk2*^*−/−*^ -double knockouts were obtained by crossing the single knockout mice. Data from different experiments was grouped randomly.

#### Generation of macrophages

Primary murine BMMs were generated by culturing bone marrow cells with M-CSF as described in our previous publication [79]. In brief, the mice were sacrificed, and bone marrow was harvested from the femur, tibia, and hip bones. The bone marrow cells were cultured in RPMI 1640 media (Gibco, Thermo-Fisher Scientific Inc) supplemented with 8% fetal bovine serum (Gibco) (R8), 50 μg/mL gentamicin (Gibco #15750060), and 5 ng/mL macrophage colony-stimulating factor (Biolegend). After 7 days, macrophages were harvested for usage.

#### Reagents

Ultrapure LPS (*E. coli* 0111: B4) was obtained from Millipore Sigma (L4524). Pan-caspase inhibitor Z-VAD-FMK was obtained from ApexBio (#A1902). Emricasan (EMR) was obtained from Selleckchem (#S7775). Recombinant mouse M-CSF (#576404), and mouse IFN-β1 (#581302), were obtained from Biolegend. Recombinant mouse TNF-alpha was obtained from R&D Systems (#410-MT-010/CF). Mouse IFN-β1 ELISA assay kit was obtained from PBL Assay Science (#124001-1). Actinomycin-D was obtained from MP Biomedicals (#02194525-CF). P38^MAPK^ inhibitor- Ralimetinib (LY2228820) dimesylate, (#S1494) and the MK2 inhibitor III (#S6930) were obtained from Selleckchem. RipK3 inhibitor GSK872 was obtained from Selleckchem (#S8465), RipK1 inhibitor II, 7-Cl-O-Nec-1 was obtained from Millipore Sigma (#5042970001).

#### Cell culture and viability

BMDMs were stimulated in 96-well tissue culture plates with LPS+zVAD (50μM) or EMR (10μM). In some experiments, BMDM were co-treated with various inhibitors and agonists before stimulation and incubated for specified time points before testing the cell viability or collecting protein lysates. Cell viability was measured using a 3-[4,5-dimethylthiazol-2-yl]-2,5-diphenyltetrazolium bromide (MTT) assay [80]. The MTT reagent was diluted with R8 media at a final concentration of 0.5 mg/mL and incubated at 37 °C. After 2 h, DMSO was added to solubilize MTT crystals and absorbance was measured at a wavelength of 570 nm with a reference wavelength of 650 nm on a Molecular Devices FilterMax plate reader.

Cell death was assessed by quantifying the uptake of neutral red, as described previously [15, 81]. In brief, cells were incubated with neutral red dye (1:20) (Millipore Sigma #N2889) until viable cells became visibly red. The cells were then washed once with PBS to remove any free dye, and the cells were lysed with a solubilization solution to release the dye that had accumulated within live cells. The absorbance of the solubilized dye was quantified by colorimetric analysis at 570 nm on a FilterMax F5 microplate reader (Molecular Devices).

#### Cell imaging

For cell imaging, macrophages were plated in a 96-well flat-bottom plate at a density of 1 × 10^5^ cells per well 24 h before the addition of inhibitors and agonists. Cells were treated with corresponding reagents for a specific time period, stained with Hoechst (2.5 μg/mL; Invitrogen, 33342) and propidium iodide (1:10 dilution; BD Pharmingen, 550825), and incubated at 37 °C for 20-30 min before evaluation by immunofluorescence microscopy using a Zeiss AxioObserver.D1 microscope. To count live versus dead cells, we employed a Python-based automated image analysis pipeline that leverages OpenCV for image processing, and NumPy for computation. Samples were converted to grayscale and binarized via Otsu’s method to highlight and distinguish features of interest from the background. External contours were calculated using OpenCV’s contour detecting algorithm, which sets to retrieve only the external contours and approximate the contour shapes to reduce the number of points. For each detected contour, the minimal enclosing circle was measured, providing the center and radius of the circular features in the sample. Each point is then calculated and circled on the original sample for manual verification.

#### Western blotting

Cells were stimulated as described above and cell extracts were reconstituted in SDS lysis buffer containing 1% β-mercaptoethanol and heated immediately at 95 °C for 5 min. Lysates were separated by SDS-PAGE and transferred to the PVDF membrane. Immunoblot analysis was performed using the following antibodies:p-RipK1^S166^ (Cell Signaling #31122), p-RipK1^S321^ (Cell Signaling #83613), RipK1 (BD Biosciences #610459), p-RipK3^T231/S232^ (Cell Signaling #91702), RipK3 (ProSci #2283), p-MLKL^S345^ (Cell Signaling #37333), MLKL (Millipore Sigma #MABC604), p-MK2^T334^ (Cell Signaling #3007), MK2 (Cell Signaling #3042), p-SAPK/JNK^T183/Y185^ (Cell Signaling #9251, SAPK/JNK (Cell Signaling #9252), p-p44/42 MAPK (Erk1/2)^T202/Y204^ (Cell Signaling #4370), p44/42 MAPK (Erk1/2) (Cell Signaling #4695), p-P38 MAPK^T180/Y182^ (Cell Signaling #9211), p38 MAPK (Cell Signaling #8690), p-P70 S6 Kinase^T389^ (Cell Signaling #9234), P70 S6 Kinase (Cell Signaling #2708), p-eIF4E^S209^ (Cell Signaling #9741), p-4E-BP1^T37/46^ (Cell Signaling #2855), 4E-BP1 (Cell Signaling #9644), p-AKT^T308^ (Cell Signaling #9275), AKT (Cell Signaling #9272), p-IKKα/β^S176/180^ (Cell Signaling #2697), IKKβ (Cell Signaling #8943), p-NF-κB^S536)^ (Cell Signaling #3033), NF-κB (Cell Signaling #8242), Tristetraprolin (TTP) (Cell Signaling #71632), Actin (Cell Signaling #3700), GAPDH (Cell Signaling #97166).

#### Cytokine measurement

Supernatants were collected from 96-well plates and the expression of cytokines was assessed using the mouse TNF-α (BD OptEIA #555268), mouse IL-6 (BD OptEIA #555240), mouse IL-10 (BD OptEIA #555252), mouse IL-12p70 (BD OptEIA #555256) and mouse IFN-β (R&D System #DY8234-05) according to the manufacturer’s instructions. The absorbance was detected at 450–570 nm on a FilterMax F5 multimode microplate reader (Molecular Devices).

The expression of IFN-I was also measured using a reporter cell line. ISRE-L929 cells were seeded at 5×10^4^ cells per well in a 96-well tissue culture plate and incubated at 37°C with 50μl of culture supernatants for 4 h. Using the luciferase assay system kit (Promega, E1500), the luminescence was measured by a Molecular Devices Emax plate reader and data were analyzed by SoftMax Pro.

#### Quantitative RT-PCR

Total RNA was extracted using the RNeasy Mini Kit (Qiagen) as per the manufacturer’s instructions. cDNA synthesis was performed using the iScript cDNA Synthesis Kit (Bio-Rad Laboratories Inc) according to the manufacturer’s instructions, and samples were stored at −20 °C until use. Quantitative real-time PCR was performed using the Bio-Rad CFX384 Touch Real-Time PCR System (Bio-Rad Laboratories Inc) in conjunction with SYBR Green PCR Master Mix (Thermo Fisher Scientific Inc).

The primers used were as follows:

*Cxcl1:* (F) 5’-TGAGCTGCGCTGTCAGTG-3’, (R) 5’-AGAAGCCAGCGTTCACCAGA-3’

*Ifnb1:* (F) 5’-ATGGTGGTCCGAGCAGAGAT-3’, (R) 5’-CCACCACTCATTCTGAGGCA-3’

*Zfp36:* (F) 5’-TTTCCCCTTCTGCCTTCTCT-3’, (R) 5’-TGGTGCTGGGGGTAGTAGAC-3’

*IL-12p35*:(F)5’-ATGTGTCAATCACGCTACCTCC-3’,(R)5’TCAGGCGGAGCTCAGATAGCC-3’

*IL-12p40*: (F) 5’-GTCCTCAGAAGCTAACCATCTCC-3’

(R) 5’-CCAGAGCCTATGACTCCATGTC-3’

*TNF-α*: (F) 5’-GAGAAGTTCCCAAATGGCCTCCC-3’

(R) 5’-GTATGAGATAGCAAATCGGCTGACGC-3’

*Actin*: (F) 5’-GATCAAGATCATTGCTCCTCCTG-3’

(R) 5’-AGGGTGTAAAACGCAGCTCA-3’

#### Gene expression profiling and analysis

Bone marrow-derived macrophages were treated with IFNβ (10 ng/mL) or LPS (10 ng/mL) with or without zVAD (50μM). Cells were harvested 6 h post-treatment and RNA was collected using the RNeasy Mini Kit (Qiagen). Reverse transcription of total RNA (200 ng) was conducted using the Agilent Low-input Quick Amp single color labeling kit. Labeled cRNA was hybridized to Agilent-028005 SurePrint G3 Mouse GE 8x60K Microarray (GPL10787). Exported probe data was filtered using a script in R (4.3.3). Probes with expression greater than background in at least 50% of arrays were retained. The background-subtracted data was quantile-normalized and probes representing low-expressed genes were removed from the analysis. Differential expression testing was performed in R (4.3.3) by considering genotype and treatment in the differential expression analysis design. The p-value was evaluated using the Benjamini–Hochberg procedure.”

Gene set enrichment analysis (GSEA*)* was conducted with all differentially expressed genes, using the R clusterProfiler package with the parameters minGSSize = 10, maxGSSize = 6000, pvalueCutoff = 1.0, pAdjustMethod = ‘BH’, and Eps = 0. The p-value was adjusted using the Benjamini–Hochberg procedure. Gene sets with adj.Pval < 0.05 were considered significant if the NES was > 1.5, or < −1.5. The gene sets that are most significantly enriched in WT cells upon necroptosis induction (‘TNFα signaling pathway’ and ‘Inflammatory Response LPS’) were selected for further analysis. Gene set enrichment plots were used to determine the leading-edge genes enriched in each pathway. Heatmaps of the leading-edge genes were generated using median-centered log_2_FC differential expression values.

#### Analysis of ChiP-Seq data

ChIP-seq data was retrieved from the NCBI SRA (GSE115435) [49] and decompressed into a fastq file format using fasterq-dump. Adapter contamination and low-quality sections of the raw reads were removed using fastp. The trimmed reads were aligned on the Mus musculus mm10 genome using the STAR aligner. Duplicate read pairs were marked using Picard and replicates were merged into a single bam file using samtools. SSP was utilized to determine chromatin fragment length and bamCoverage was used to calculate read density. The resulting bigWig files were loaded on the integrative genomics viewer for analysis.

#### Statistical analyses

All graphs show the average results taken from at least three independent experiments. Error bars show the standard error of the mean, and statistical significance between groups was determined by using the student’s *t*-test or ANOVA using the GraphPad Prism 10 software. The Brown–Forsythe test used to test for equality of variances. Statistical evaluation of GSEA and differential gene expression (DGE) was performed in R. Specifically, the calculated P-values following DGE analysis and GSEA were adjusted using the Benjamini–Hochberg (B-H) procedure.

### Supplementary information

Supplementary Figure 1

Supplementary Figure 2

Supplementary Figure 3

Supplementary Figure 4

Supplementary Figure 5

Supplementary Figure 6

Supplementary figure legends

Supplementary material

## Data Availability

The authors declare that all other data supporting the findings of this study are available within the article and its supplementary information files. All the original highthroughput microarray data has deposited at GEO (GSE134549).
