# Supplementary figures and images for: Regulation of Zfp36 by ISGF3 and MK2 restricts the expression of inflammatory cytokines during necroptosis stimulation
Source: Cell Death Dis. 2024 Aug 8;15(8):574. doi: 10.1038/s41419-024-06964-4 (PMC11310327; doi:10.1038/s41419-024-06964-4)

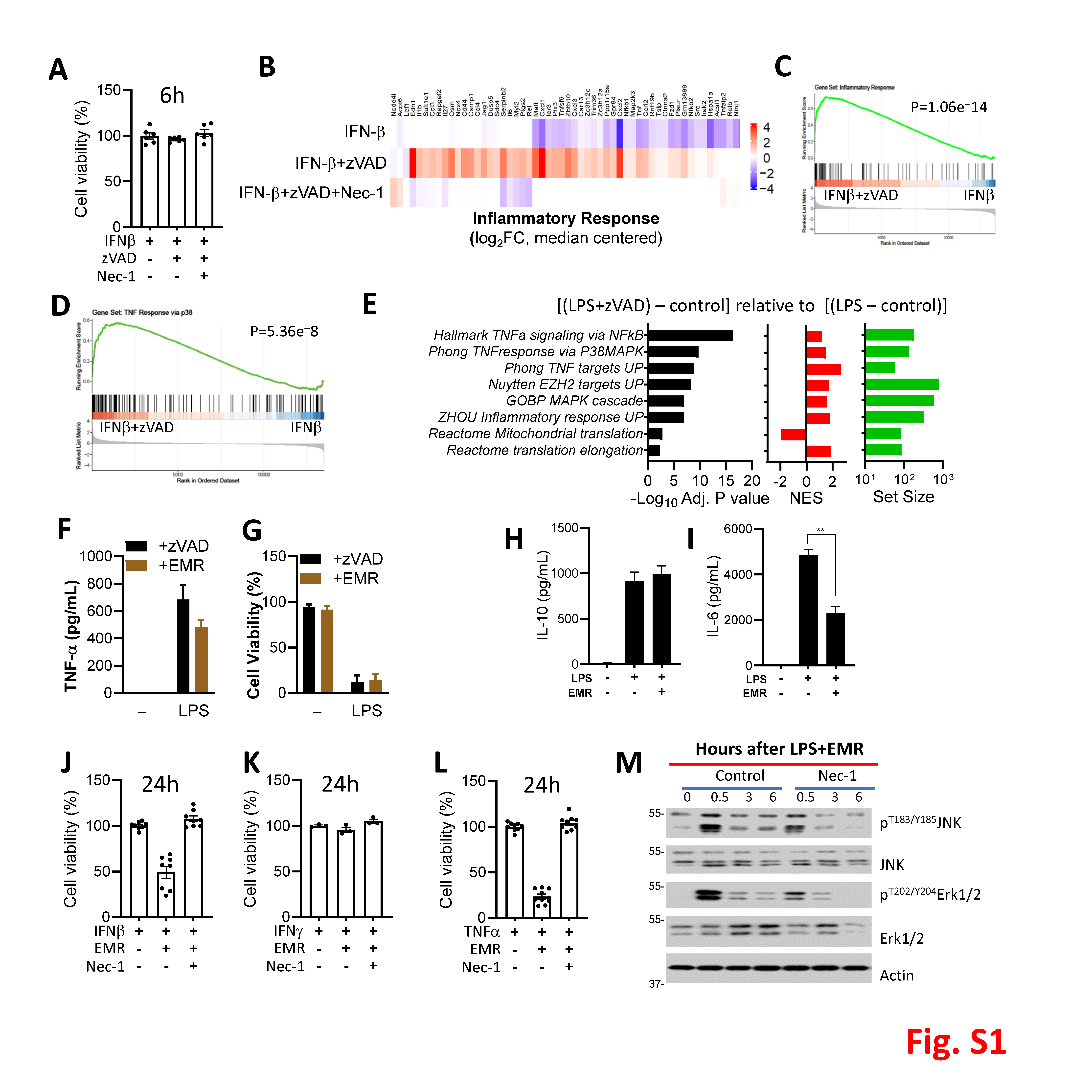

Supplement: Supplementary file 1 — Supplementary Figure 1 [file 41419_2024_6964_MOESM1_ESM.tif]

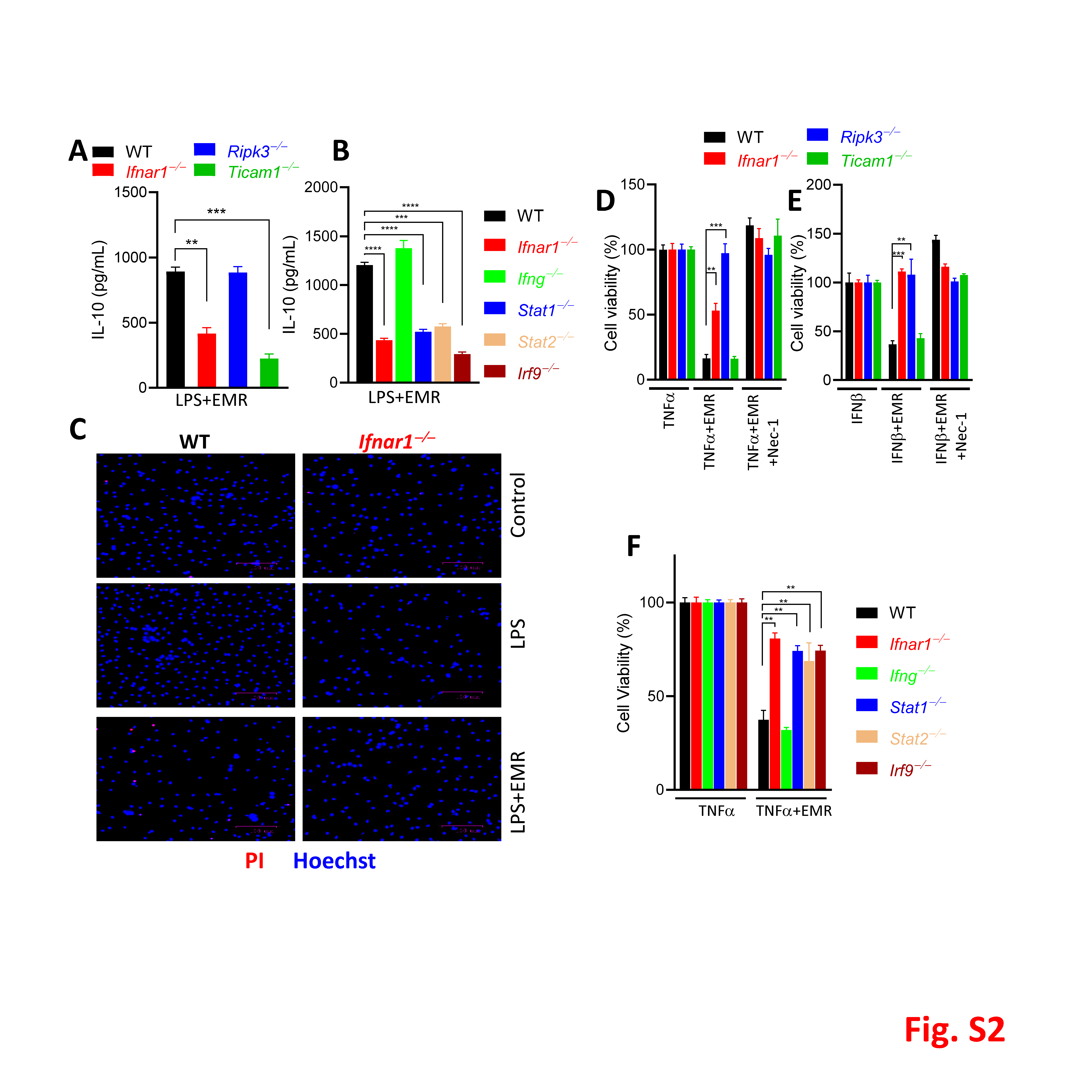

Supplement: Supplementary file 2 — Supplementary Figure 2 [file 41419_2024_6964_MOESM2_ESM.tif]

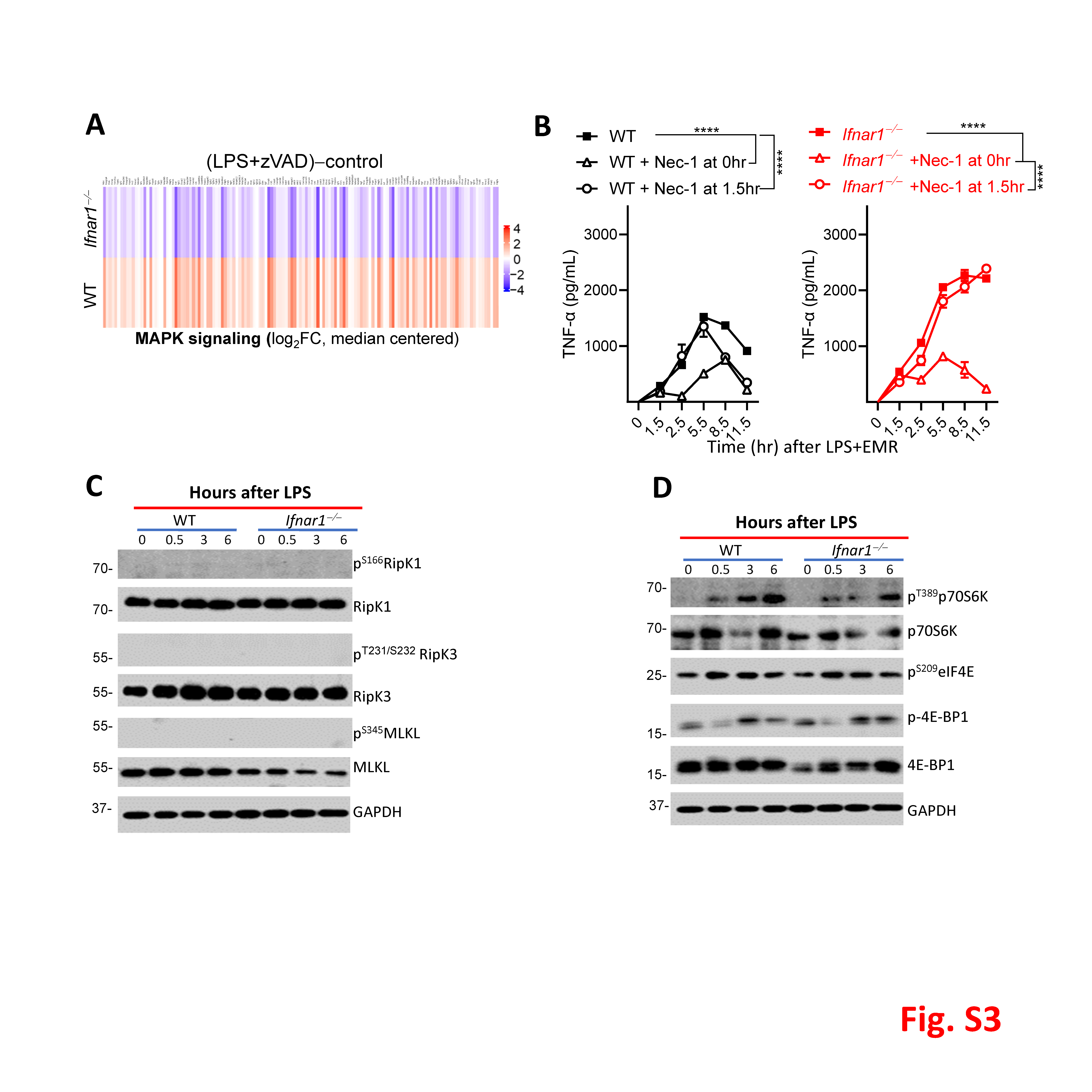

Supplement: Supplementary file 3 — Supplementary Figure 3 [file 41419_2024_6964_MOESM3_ESM.tif]

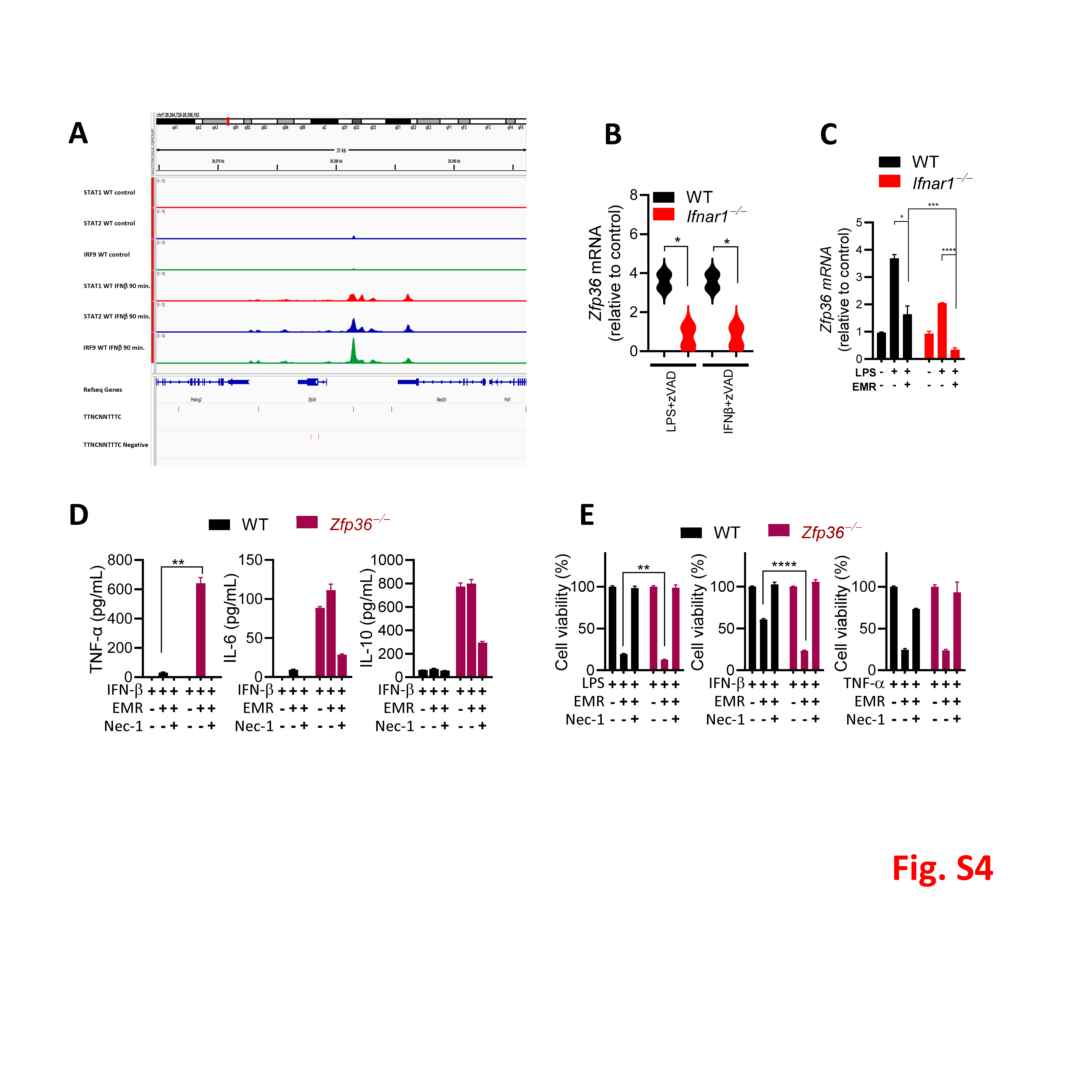

Supplement: Supplementary file 4 — Supplementary Figure 4 [file 41419_2024_6964_MOESM4_ESM.tif]

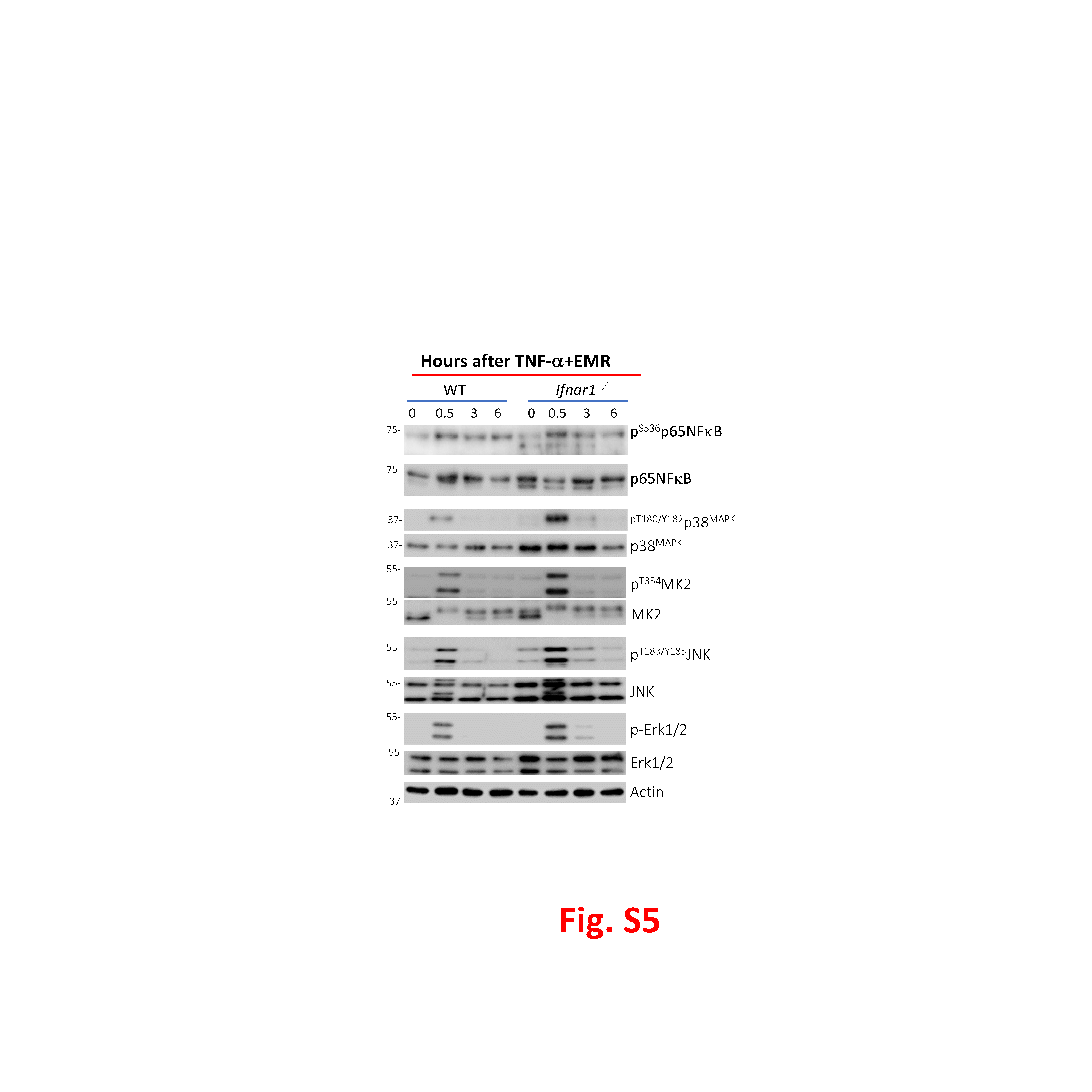

Supplement: Supplementary file 5 — Supplementary Figure 5 [file 41419_2024_6964_MOESM5_ESM.tif]

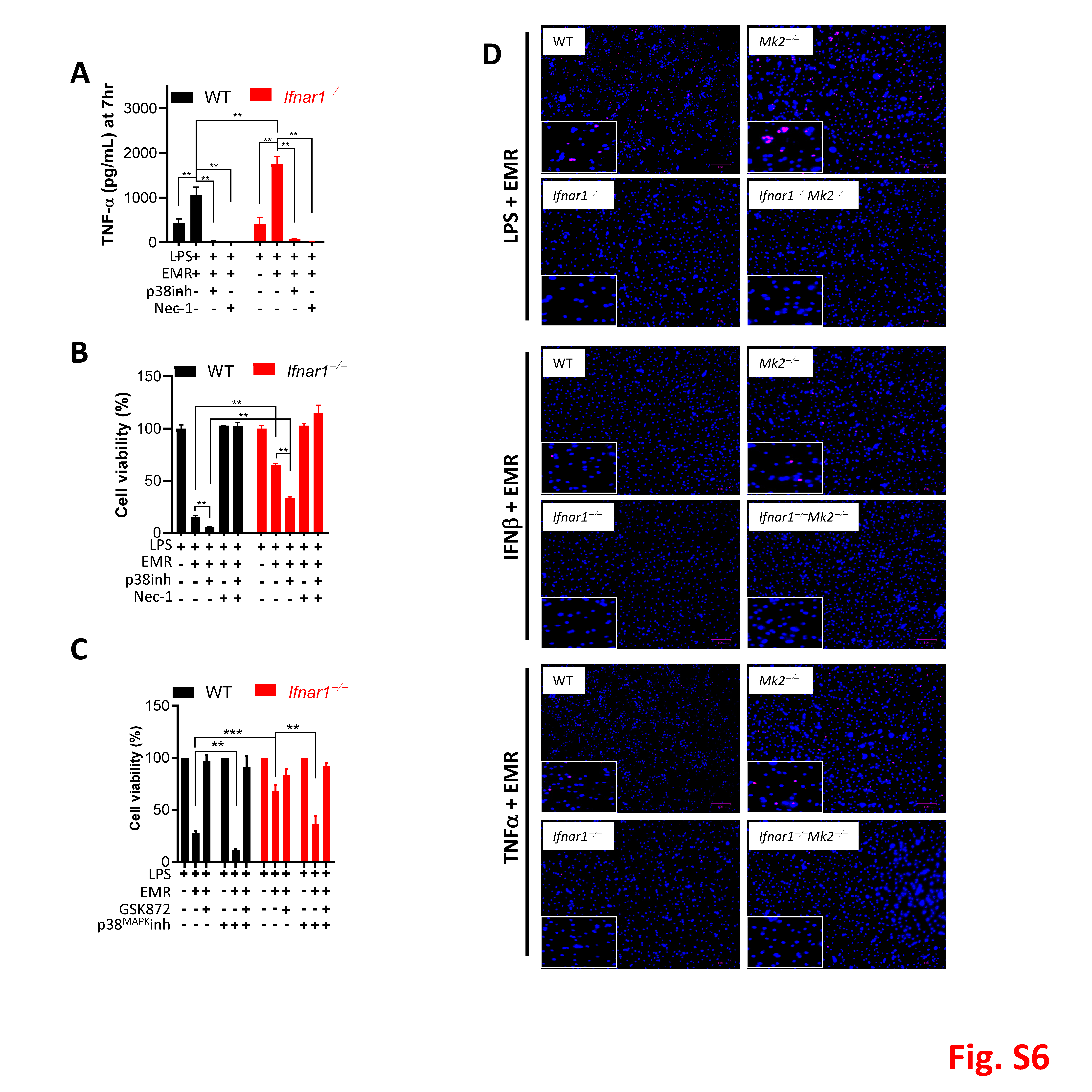

Supplement: Supplementary file 6 — Supplementary Figure 6 [file 41419_2024_6964_MOESM6_ESM.tif]
