# Supplementary material for: Regulation of Zfp36 by ISGF3 and MK2 restricts the expression of inflammatory cytokines during necroptosis stimulation
Source: Cell Death Dis. 2024 Aug 8;15(8):574. doi: 10.1038/s41419-024-06964-4 (PMC11310327; doi:10.1038/s41419-024-06964-4)
Supplement: Supplementary file 7 — Supplementary figure legends [file 41419_2024_6964_MOESM7_ESM.docx]

**Supplementary figure legends**

**Figure S1: Necrosome activation causes upregulation of inflammatory response.**

**(A-D)** WT BMDMs were treated with IFNβ (100U/mL), zVAD-fmk (50µM), and Nec1-s (10 µM). Cell death was evaluated at 6 h by MTT (**A**). (**B-E**) Differential gene expression and GSEA was performed in BMDMs stimulated with IFNβ (100U/mL), zVAD-fmk (50µM), and Nec1-s (10 µM) (**B-D**), or LPS (1 ng/mL), and zVAD-fmk (50µM) **(E**). **(F, G)** Expression of TNFα was measured by ELISA in the supernatants collected at 6h post stimulation of WT BMDMs with LPS (1ng/mL)+zVAD (50µM) or LPS (1ng/mL)+EMR (10µM) (**F**). Cell death was measured by MTT assay at 24h post stimulation (**G**). **(H, I)** Expression of IL-10 and IL-6 was measured in the supernatants of WT BMDMs at 7 h post stimulation of WT BMDMs with LPS (1ng/mL)+EMR (10µM). (**J-L**) WT BMDMs were treated with IFNβ (100U/mL) (**J**), IFN-γ (50 ng/mL) (**K**), or TNFα (50 ng/mL) (**L**), and EMR (10µM), and Nec1-s (10µM). Cell viability was evaluated by the MTT assay at 24 h post stimulation. **(M)** Western blot analysis was performed in cell extracts collected from WT BMDMs at different time intervals post stimulation with LPS (1ng/mL)+EMR (10µM)+/- Nec1-s (10 µM). Each experiment was repeated at least three times. (***P<*0.01).

**Figure S2. ISGF3 promotes necroptosis and IL-10 expression.**

**(A-C)** BMDMs of various genotypes were treated with LPS (1ng/mL) and EMR (10µM). Expression of IL-10 was measured in cell supernatants collected at 7 h post activation (**A, B**). Cell viability was evaluated at 6 h post activation by staining with PI and Hoechst (**C**). **(D-F)** BMDMs of various genotypes were treated with TNFα (50ng/ml), IFNβ (100U/mL), EMR (10µM), and Nec-1 (10µM). Cell death was measured by MTT assay at 24 h. Each experiment was repeated at least three times. (***P<*0.01, ****P<*0.001, *****P<*0.0001).

**Figure S3. RipK1 has differential impact on TNFα expression by WT versus *Ifnar1^−/−^* cells.**

**(A)** WT and *Ifnar1^−/−^* BMDMs were treated with LPS (1ng/mL) and zVAD-fmk (50µM) for 6 h. Heatmap shows the genes in the MAPK-pathway that are downregulated in *Ifnar1^−/−^*cells. **(B)** Expression of TNFα in the supernatants collected at different time intervals following treatment of WT and *Ifnar1^−/−^* BMDMs with LPS (1ng/mL) and EMR (10µM). Nec1-s (10 µM) was added at the time or at 90 minutes post treatment with LPS+EMR. **(C, D)** Western blot analysis was performed in the cell lysates collected from WT and *Ifnar1^−/−^* BMDMs at different time intervals after the treatment of LPS (1ng/mL). Each experiment was repeated at least three times. (*****P<*0.0001).

**Figure S4: ISGF3 promotes Zfp36 transcription.**

(**A**) Genome browser track was plotted from the ChiP-seq data retrieved from [doi.org/10.1038/s41467-019-10970-y](https://doi.org/10.1038/s41467-019-10970-y). Binding of STAT1 (red), STAT2 (blue), and IRF9 (green) is shown in untreated and IFNβ treated BMDMs at ISRE sites upstream of the *Zfp36* promoter. The integrative genomics viewer track represents a ChIP-seq experiment as described in [doi.org/10.1038/s41467-019-10970-y](https://doi.org/10.1038/s41467-019-10970-y). **(B, C)** Expression of *Zfp36* mRNA was measured in WT and *Ifnar1^−/−^* BMDMs by microarray (**B**) or by qRT-PCR (**C**) at 6 h post treatment. (**D**) WT and *Zfp36^−/−^* BMDMs were treated with IFNβ (100U/mL), EMR (10µM), and Nec1-s (10 µM). Expression of cytokines was measured in cell supernatants collected at 6h post stimulation of cells. **(E)** Cell death was measured at 24 h by MTT assay in WT and *Zfp36^−/−^* BMDMs after the treatment with LPS (1ng/mL), EMR (10µM), IFNβ (10ng/mL), or TNFα (50ng/mL). Each experiment was repeated at least three times. (**P* <0.05, ***P<*0.01, ****P<*0.001, *****P<*0.0001).

**Figure S5. Upregulation of MAPK activation in *Ifnar1^−/−^* BMDMs.**

Western blot analysis was performed in cell extracts collected from WT and *Ifnar1^−/−^* BMDMs at different time intervals after treatment with TNFα (50ng/mL) and EMR (10µM). Each experiment was repeated at least three times.

**Figure S6. Treatment with the p38MAPK inhibitor reduces TNFa expression and increases cell death of *Ifnar1^−/−^* BMDMs.**

**(A, B)** WT and *Ifnar1^−/−^* BMDMs were stimulated with LPS (1ng/mL), EMR (10µM), p38^MAPK^ inhibitor (LY2228820, 4 µM), and Nec-1 (10µM). Expression of TNFα were measured in the supernatants at 7 h by ELISA (**A**), and cell death was measured at 24 h by MTT assay (**B**). Cell viability was also measured in cells treated with the RipK3 inhibitor GSK872 (5 µM) (**C**). (**D**) WT, *Ifnar1^−/−^*, *Mk2^−/−^*, and *Ifnar1^−/−^Mk2^−/−^* BMDMs were stimulated with LPS (1ng/mL)+EMR (10µM), IFNβ (10ng/mL)+EMR (10µM), and TNFα (50ng/mL)+EMR (10µM). At 9h post stimulation, cells were stained with Hoechst and PI. Each experiment was repeated at least three times. (***P<*0.01, ****P<*0.001).
