# Supplementary material for: Regulation of Zfp36 by ISGF3 and MK2 restricts the expression of inflammatory cytokines during necroptosis stimulation
Source: Cell Death Dis. 2024 Aug 8;15(8):574. doi: 10.1038/s41419-024-06964-4 (PMC11310327; doi:10.1038/s41419-024-06964-4)

Fig. 1J panels

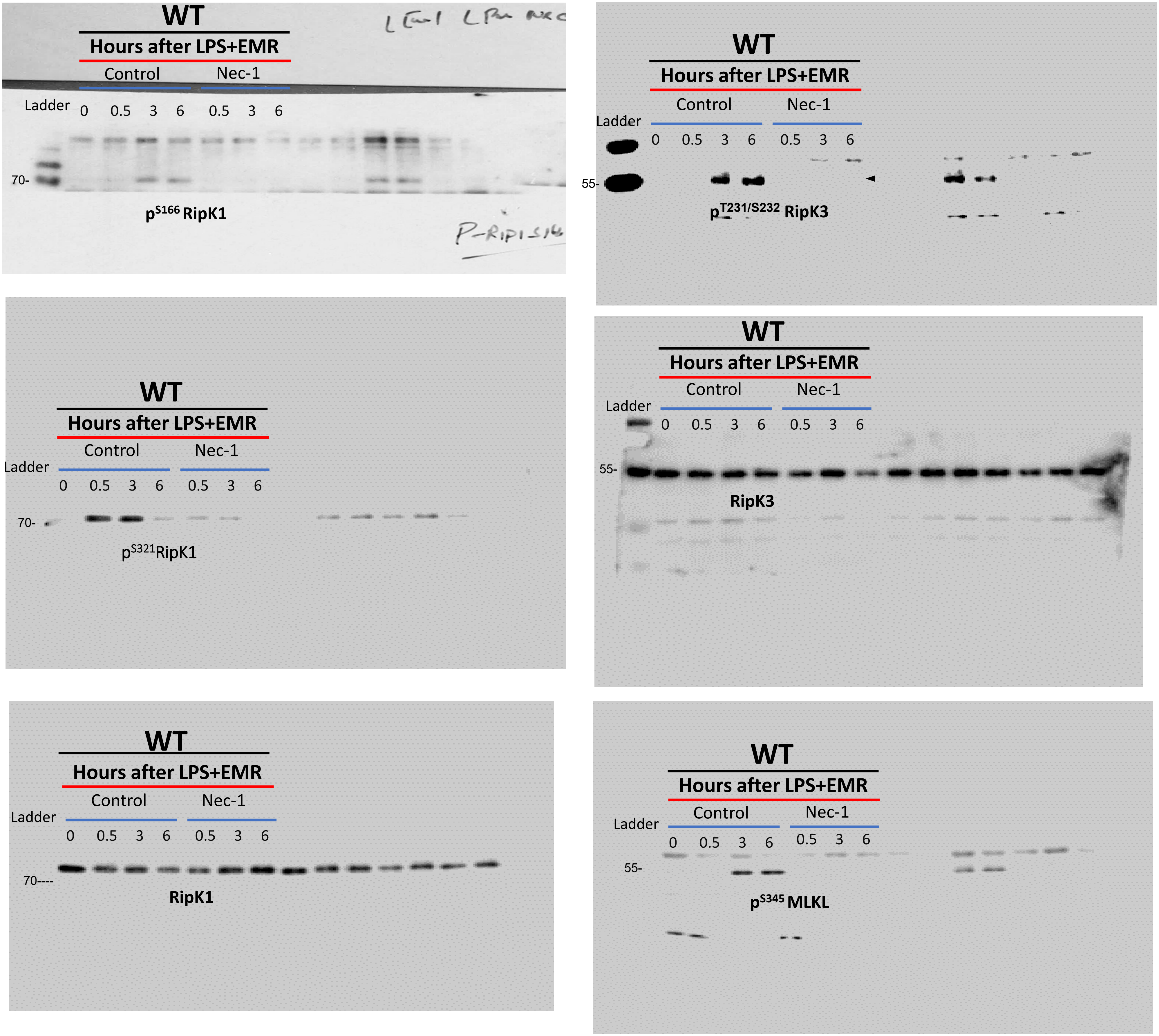

Fig. 1J panels

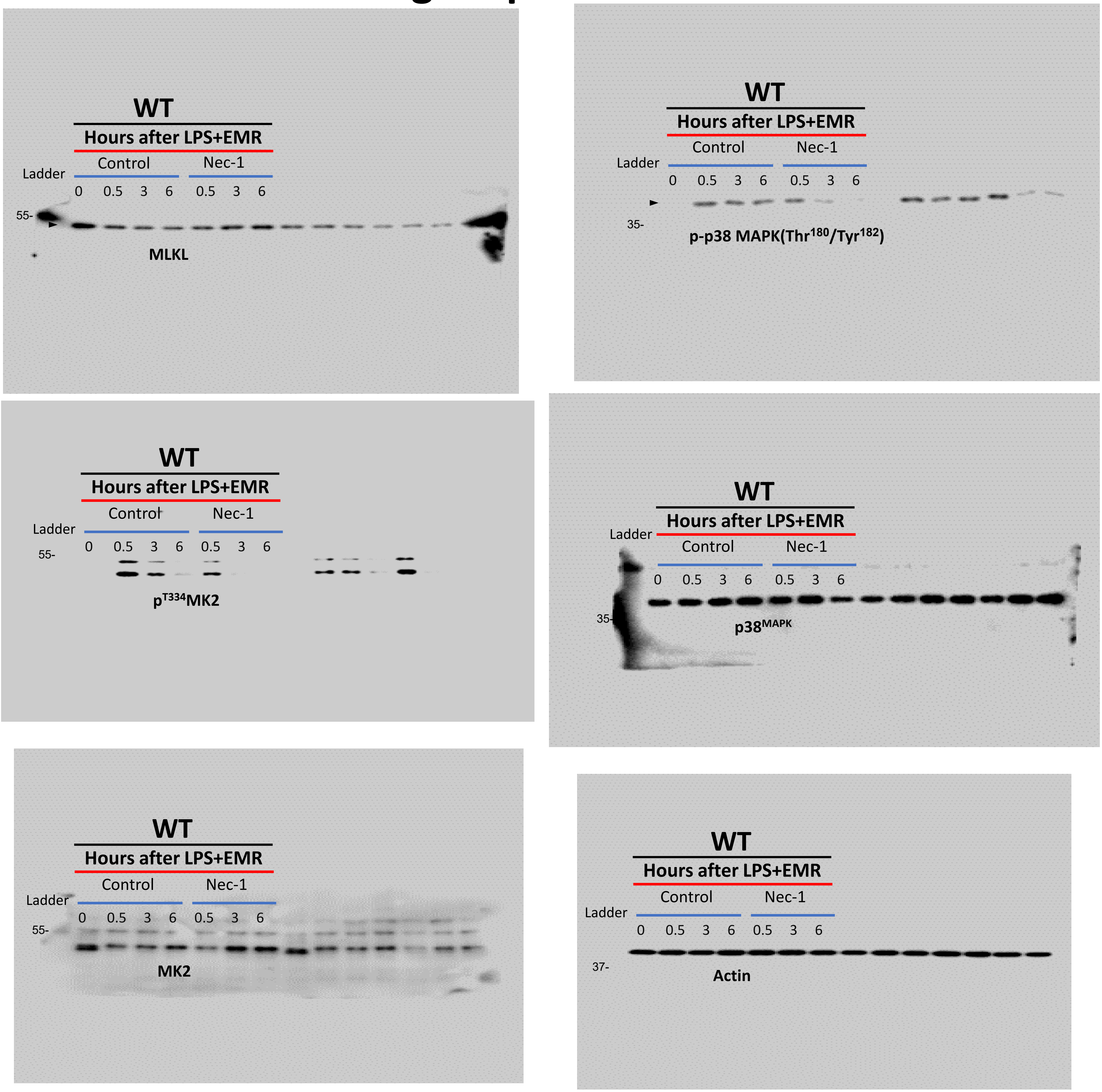

Fig. 3F panels

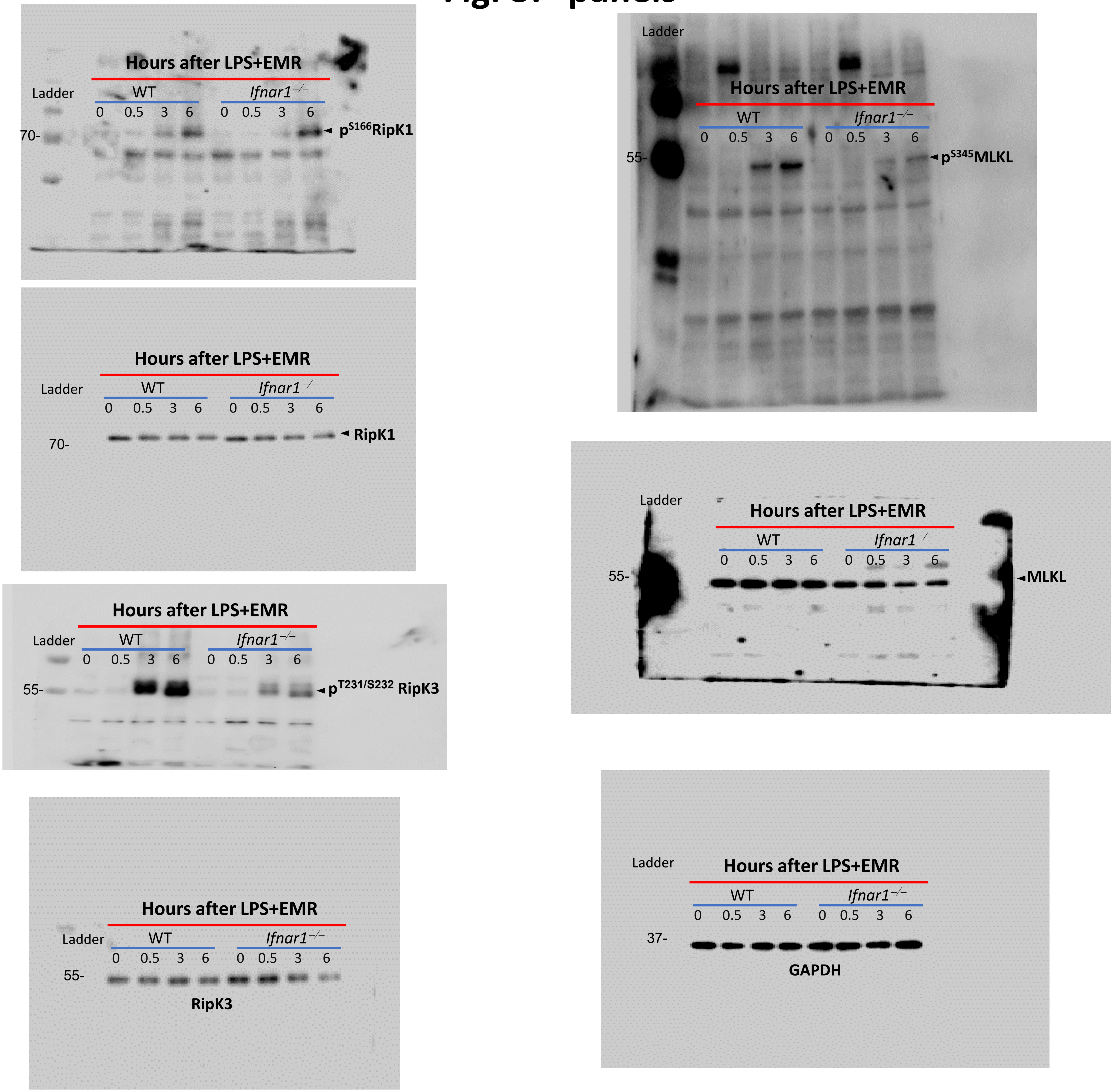

Fig. 3G panels

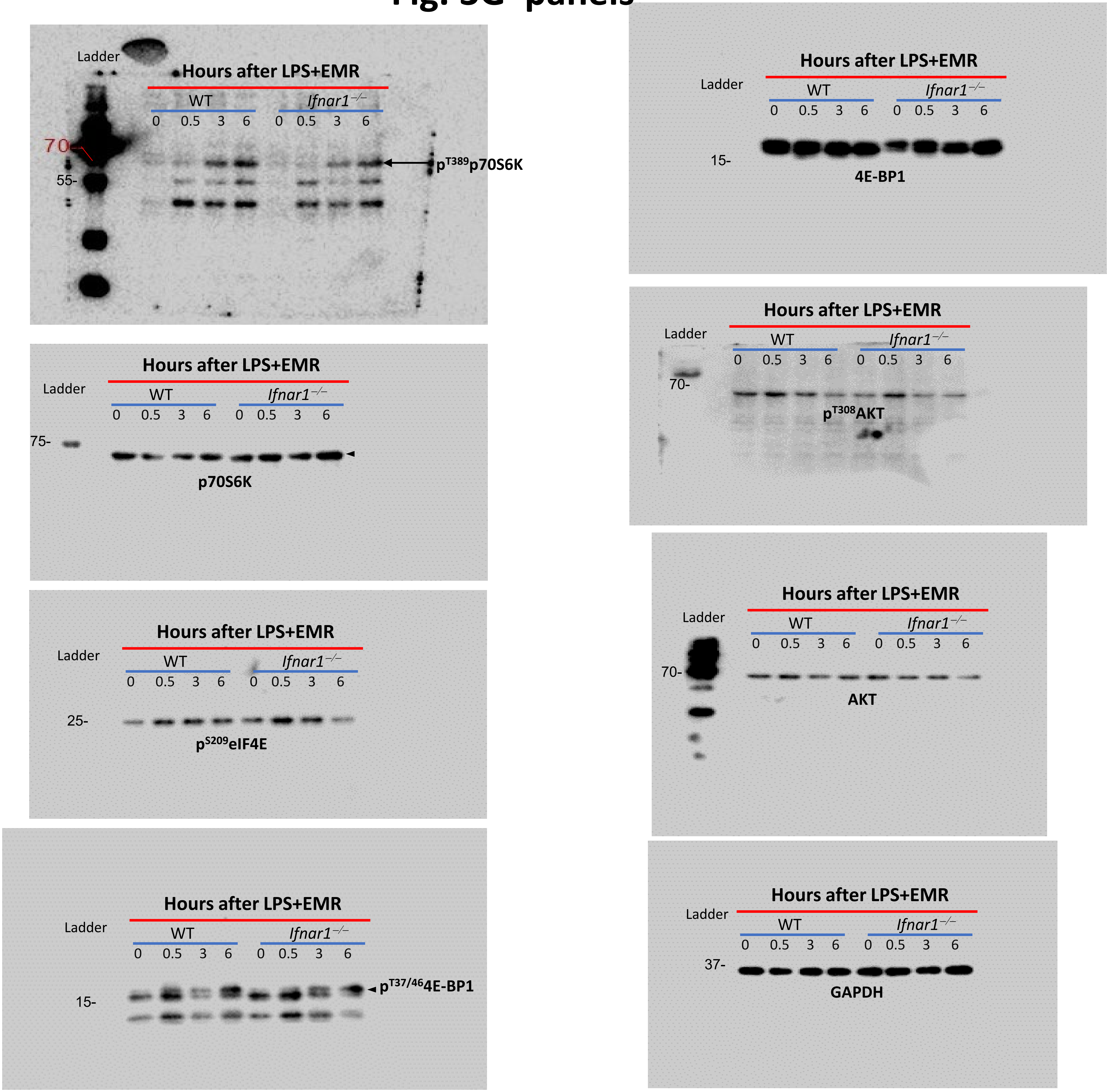

Fig. 4A panels

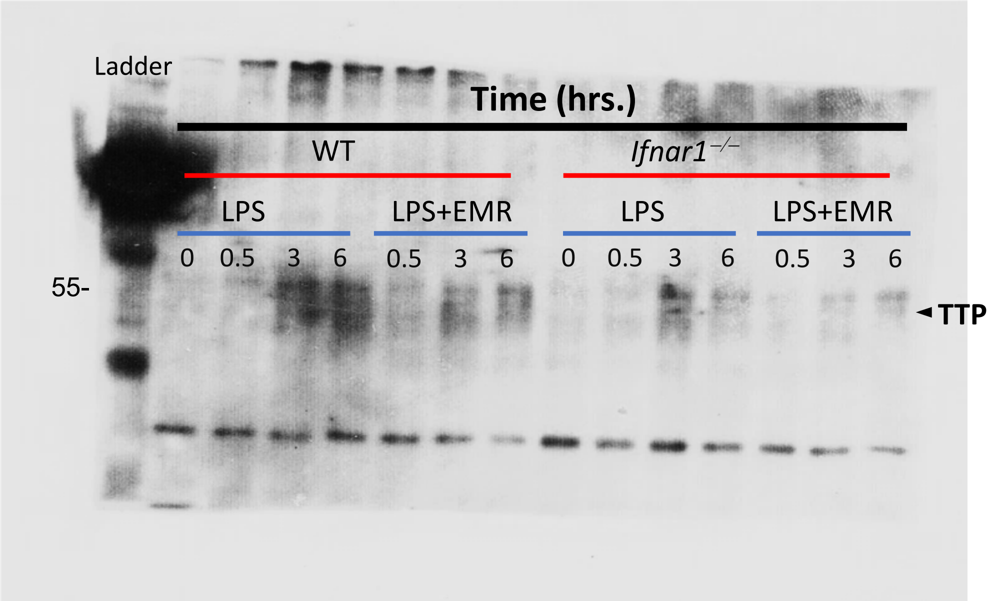

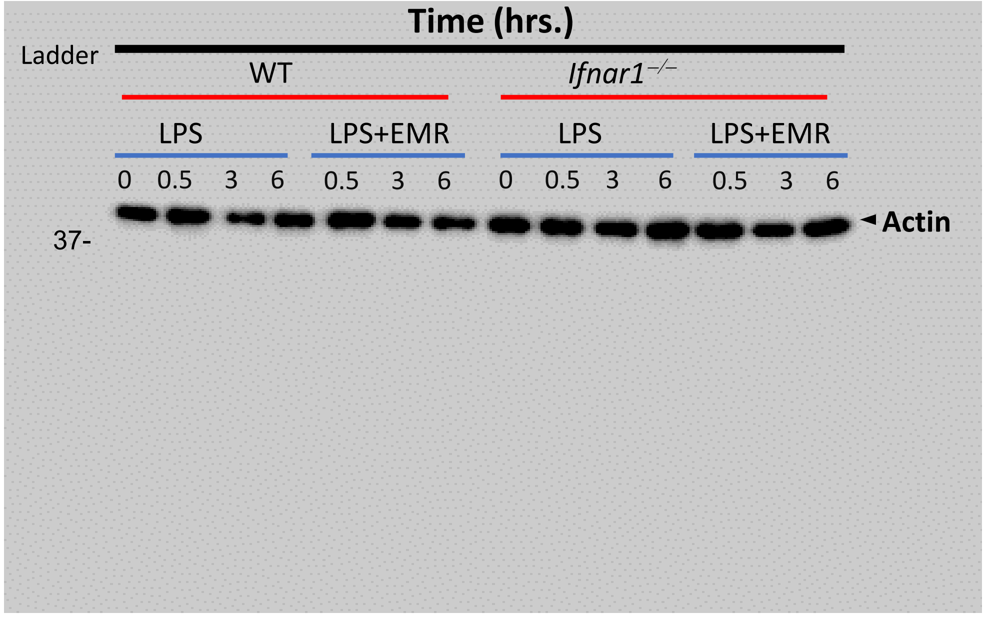

Fig. 5A panels

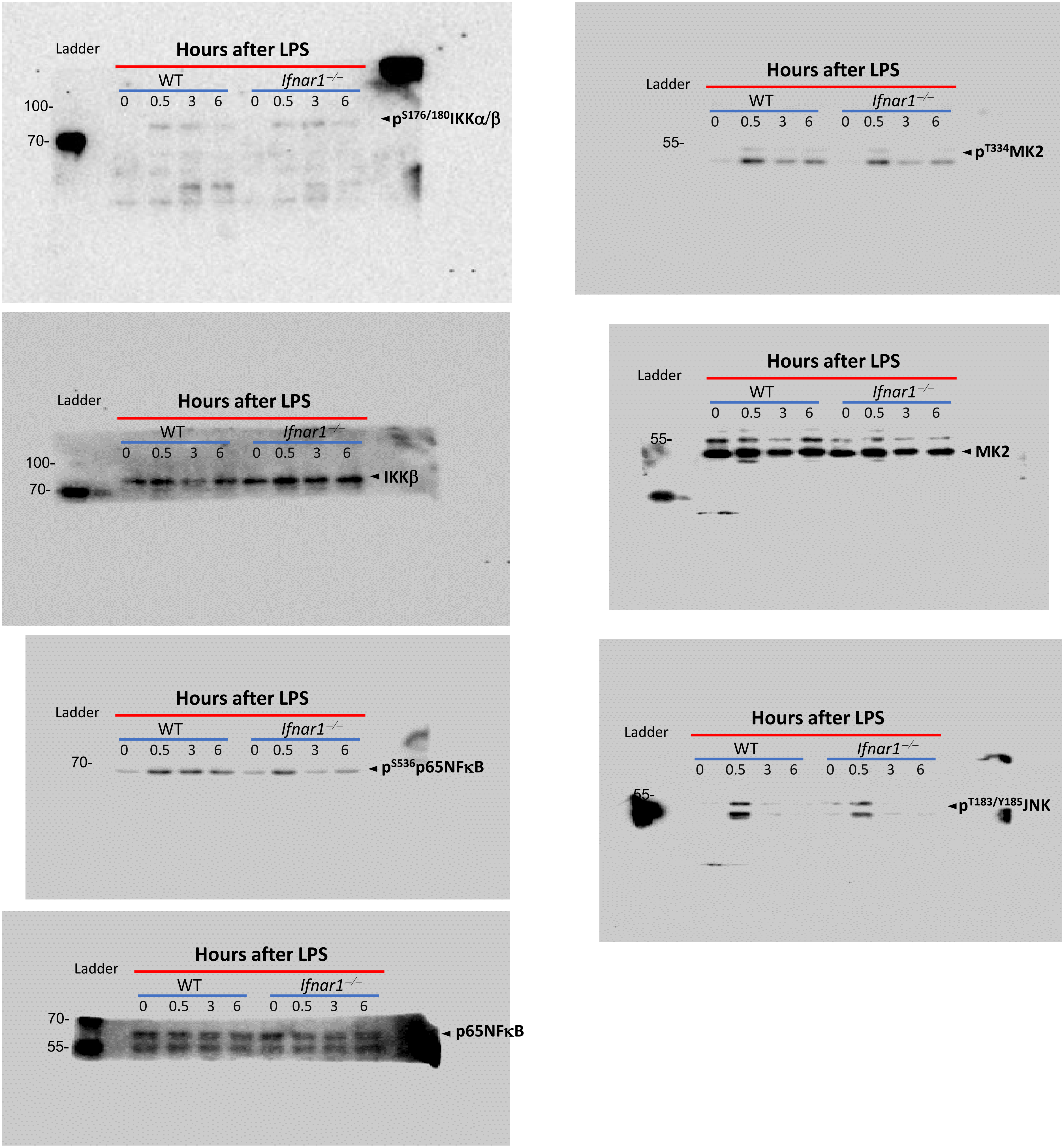

Fig. 5A panels

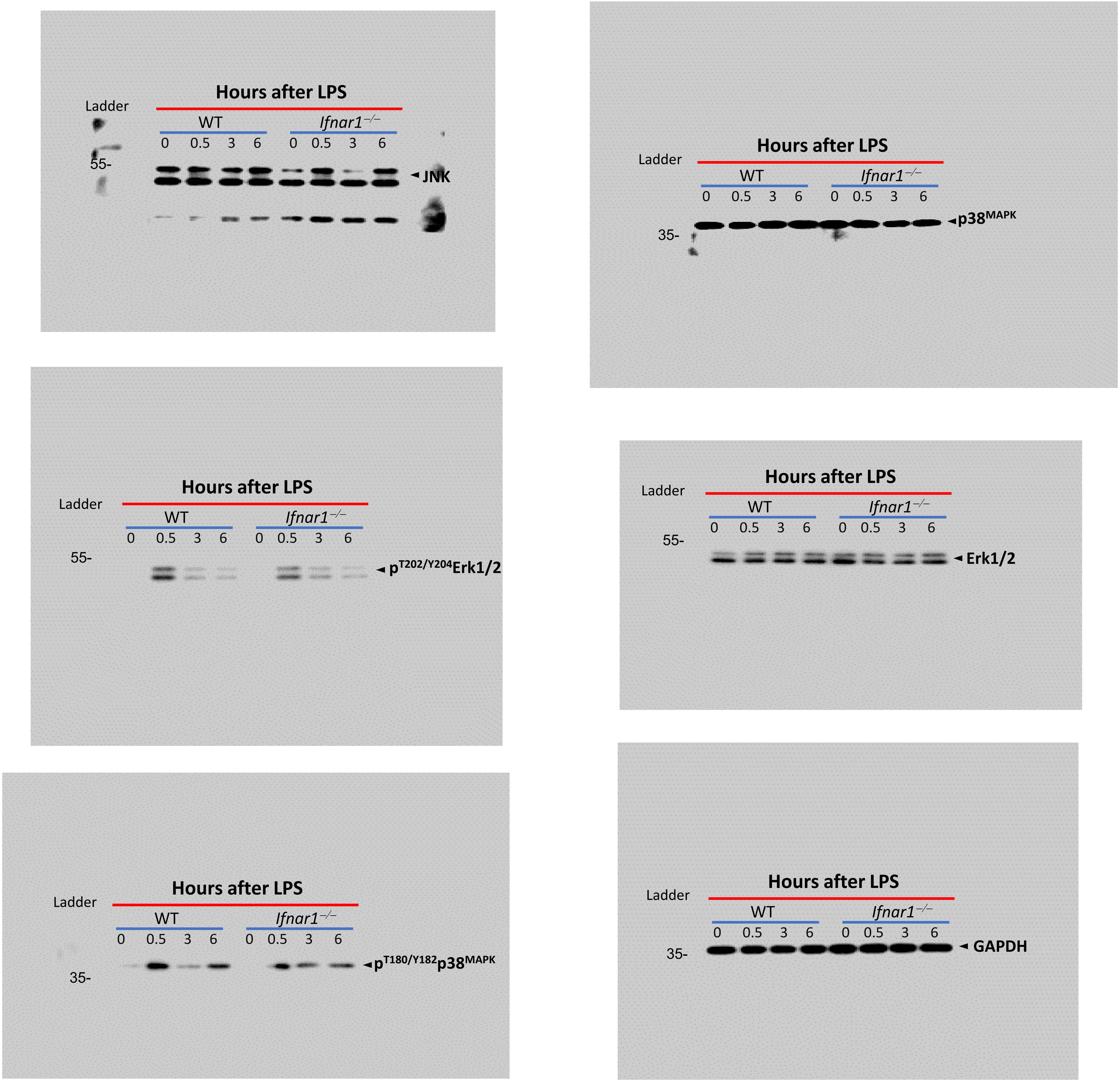

Fig. 5B panels

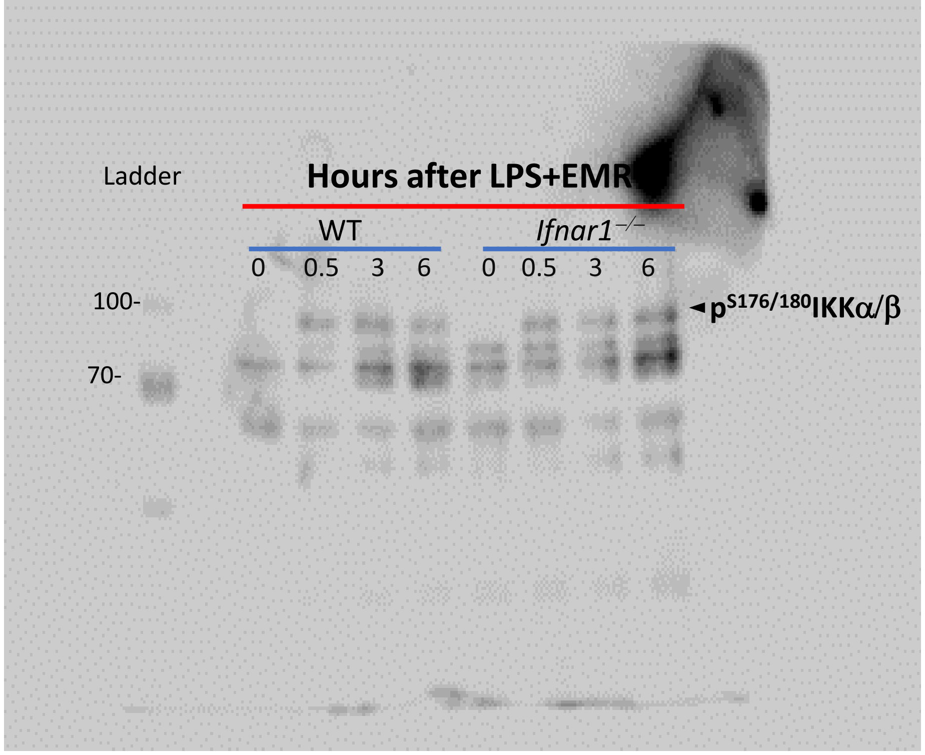

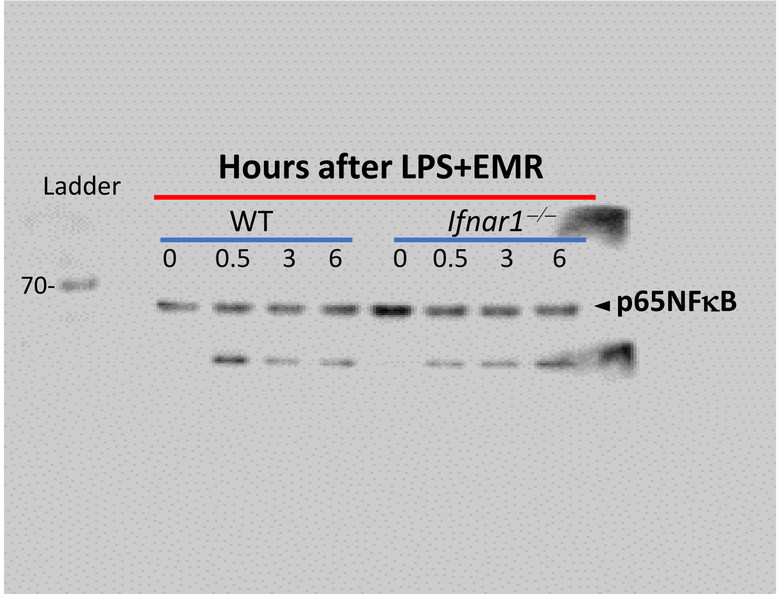

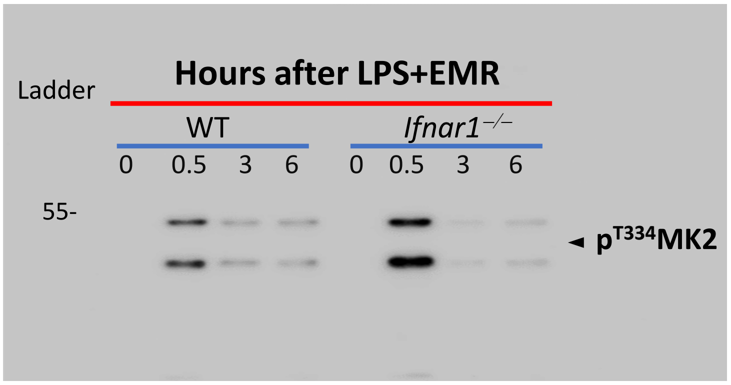

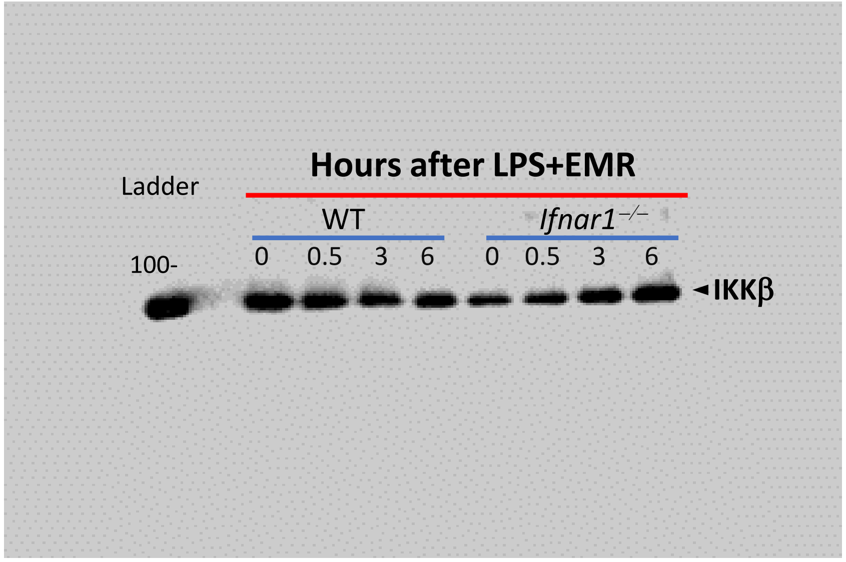

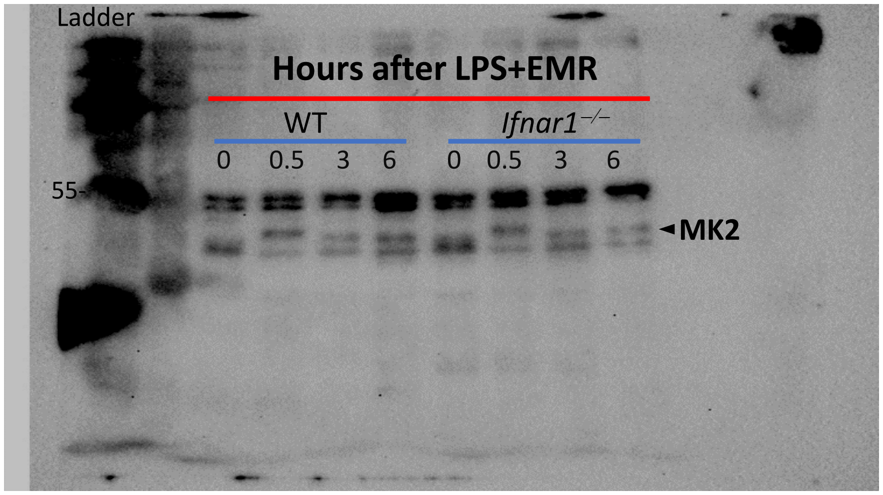

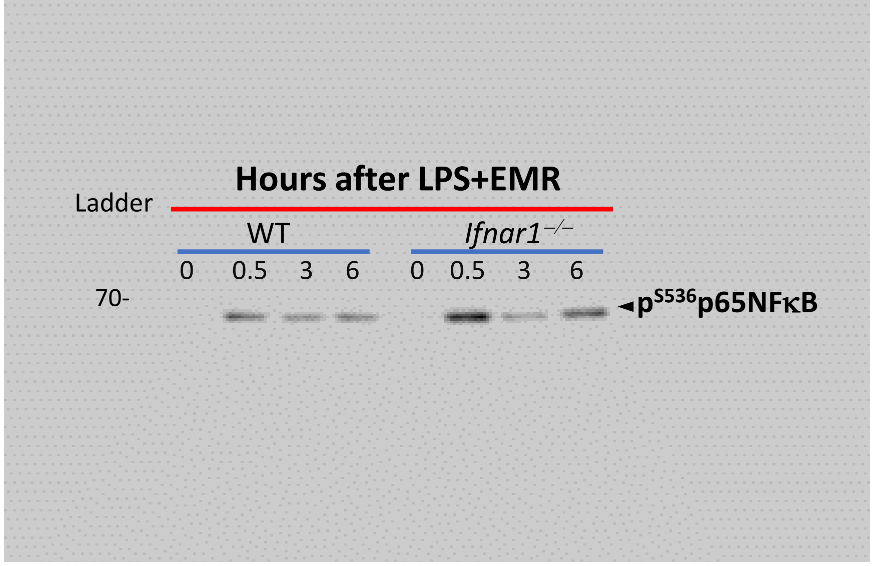

Fig. 5B panels

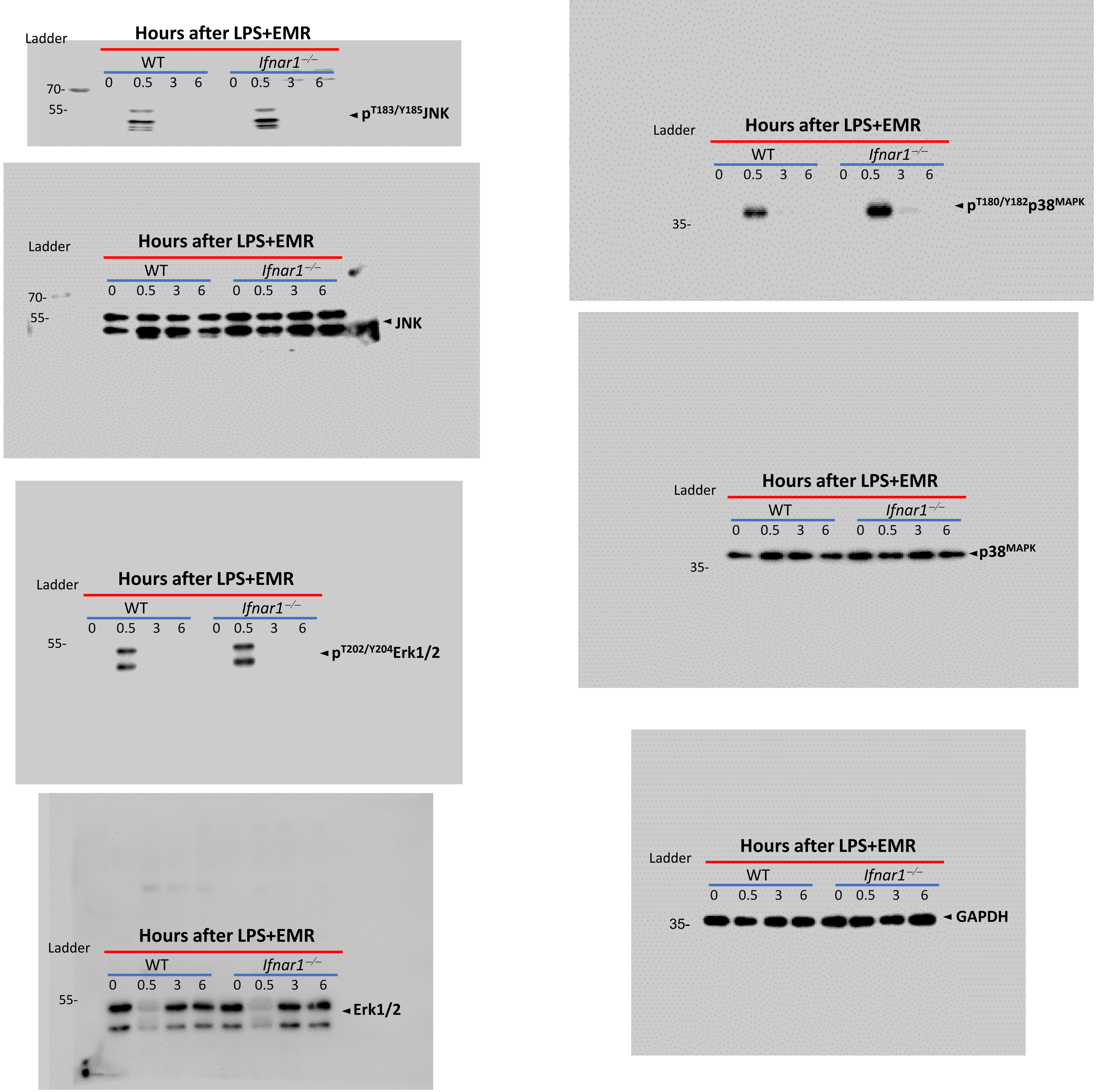

Fig. 5C panels

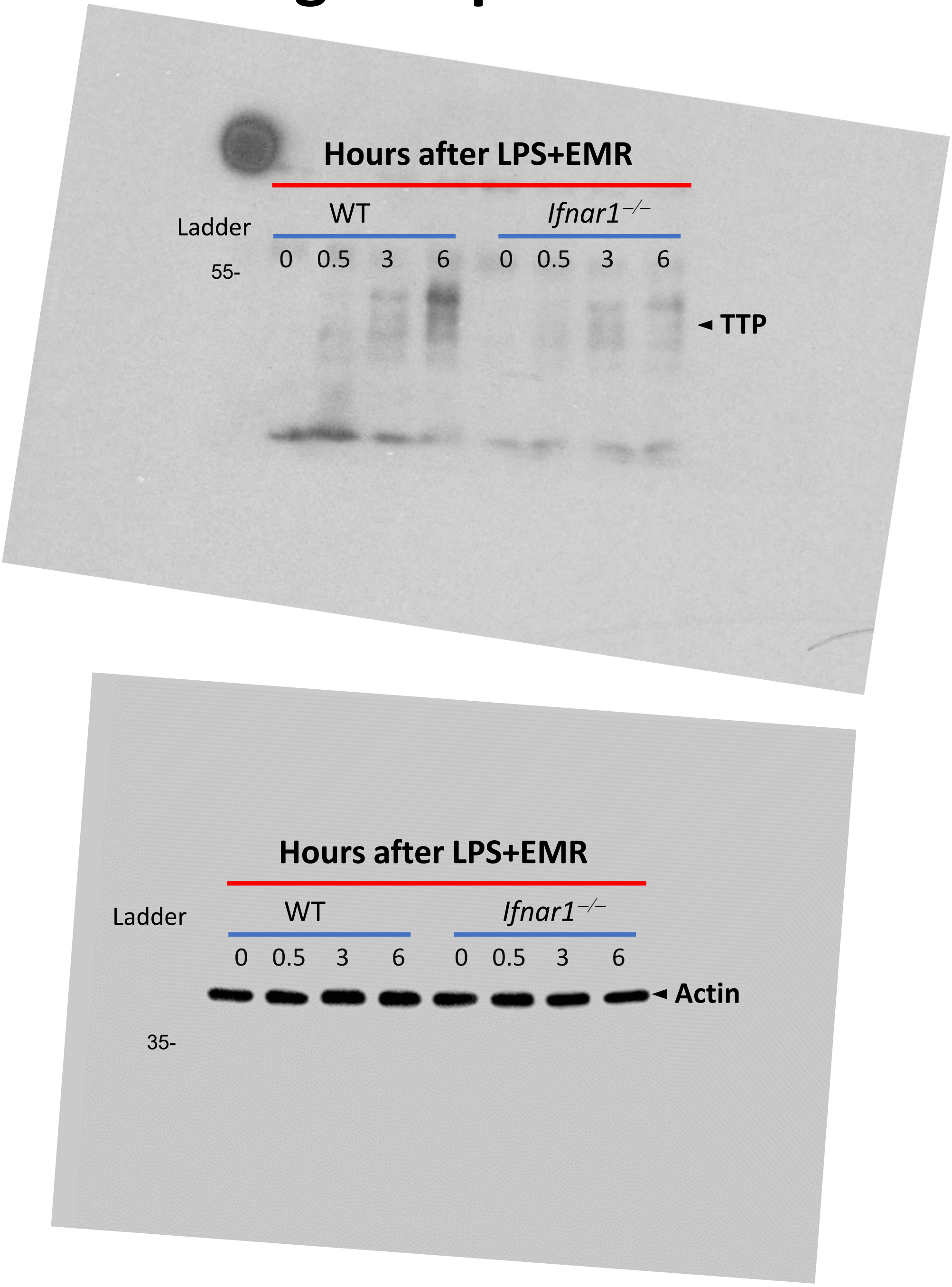

Fig. 5D panels

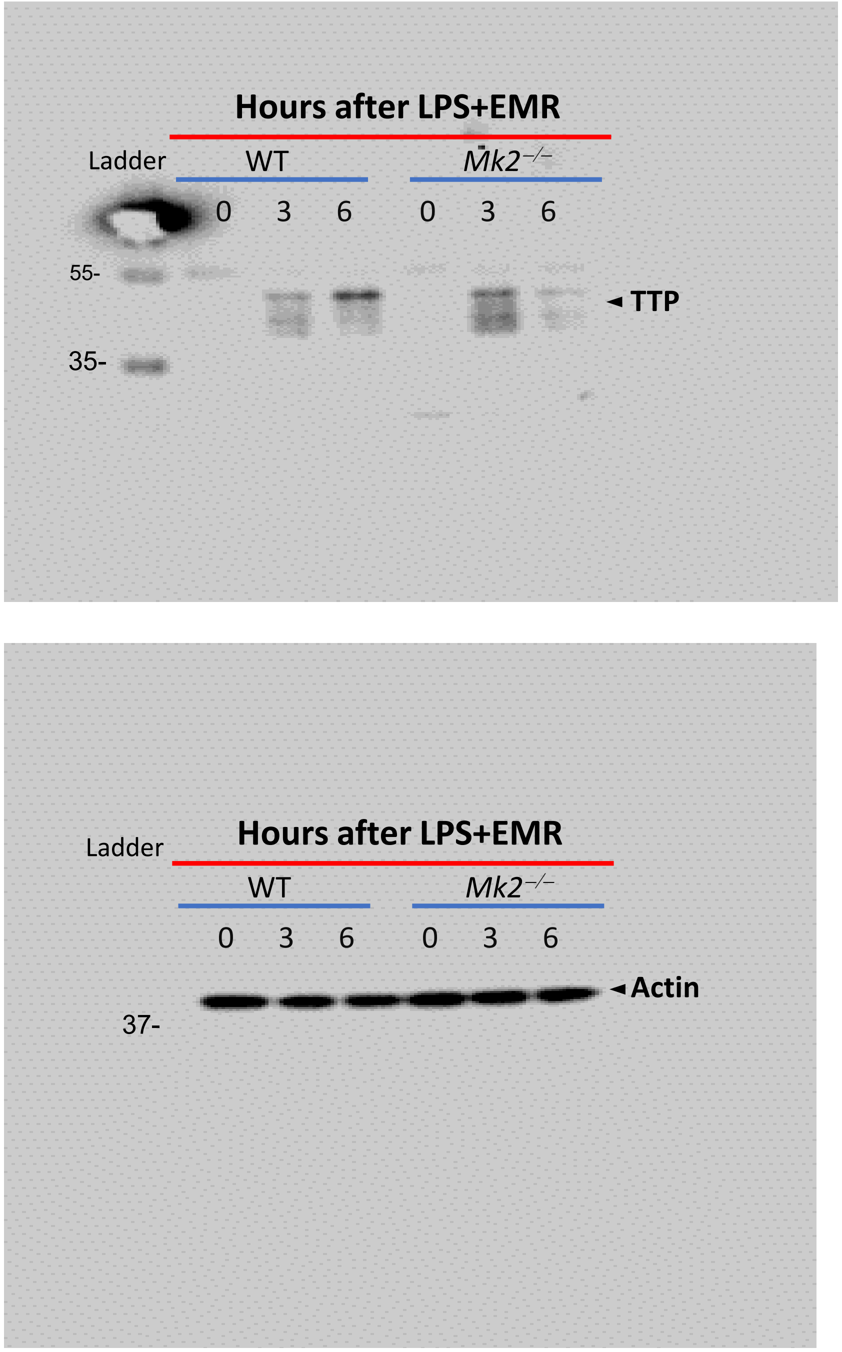

Fig. 6D panels

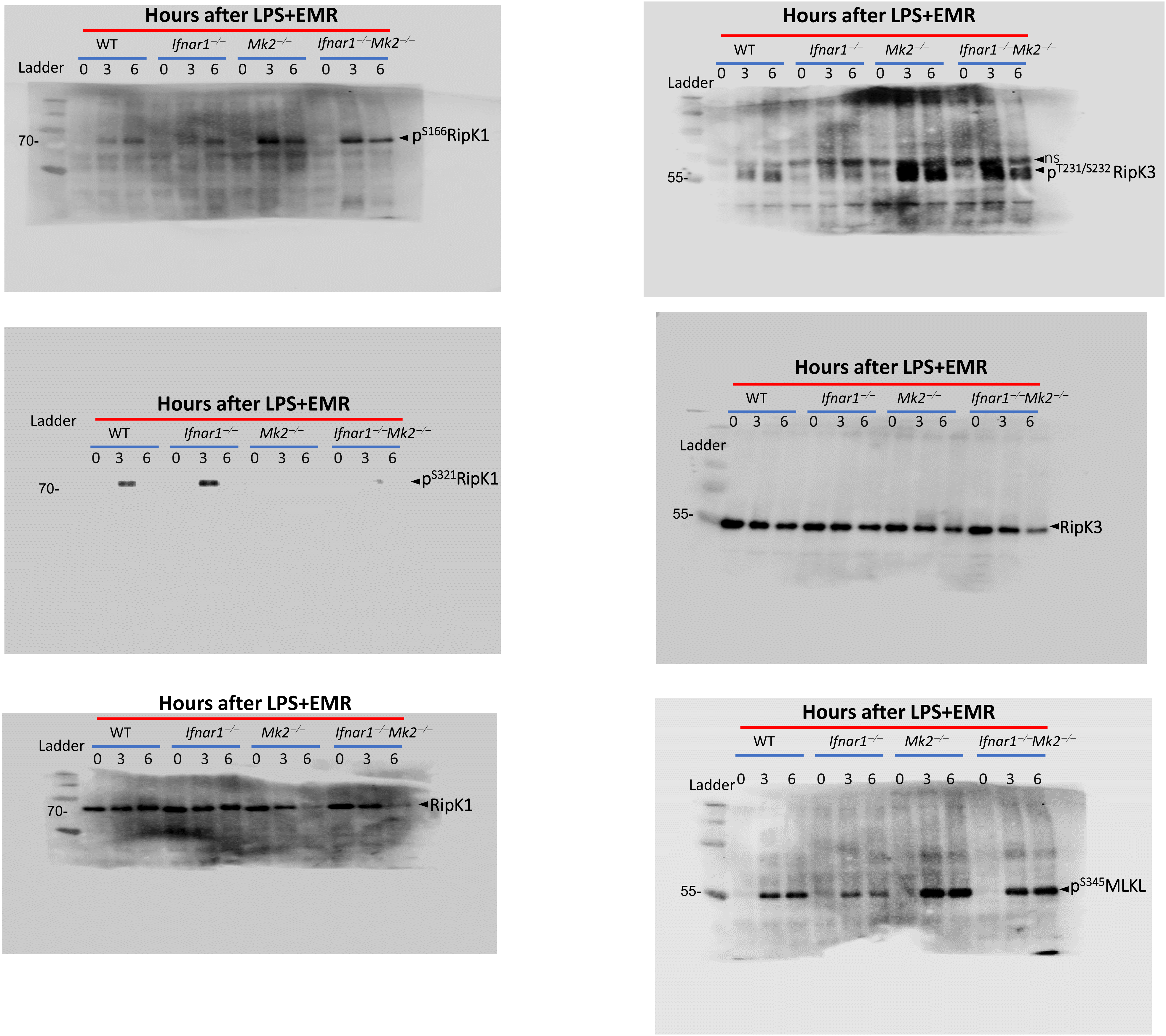

Fig. 6D panels

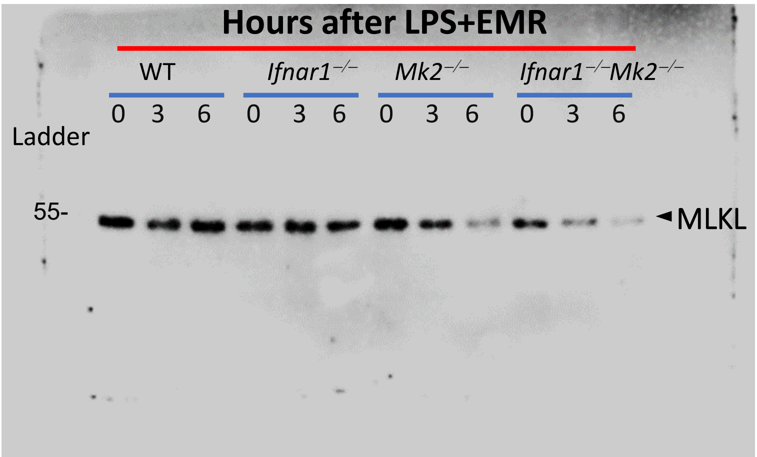

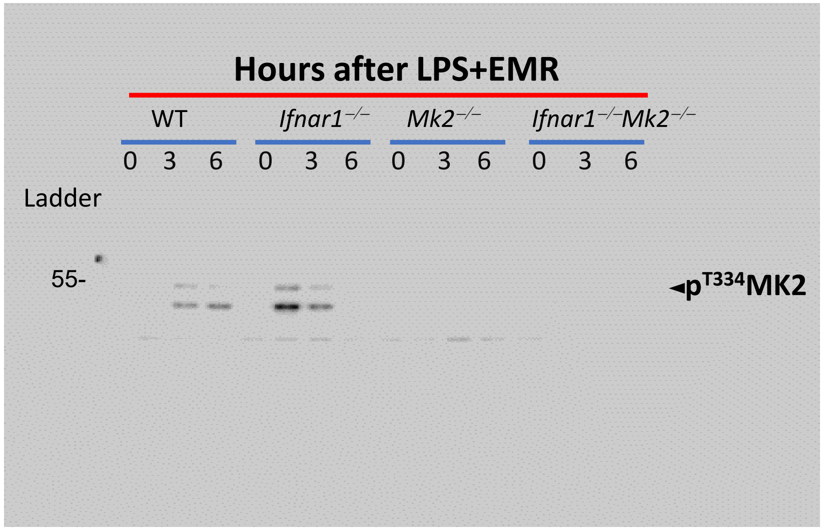

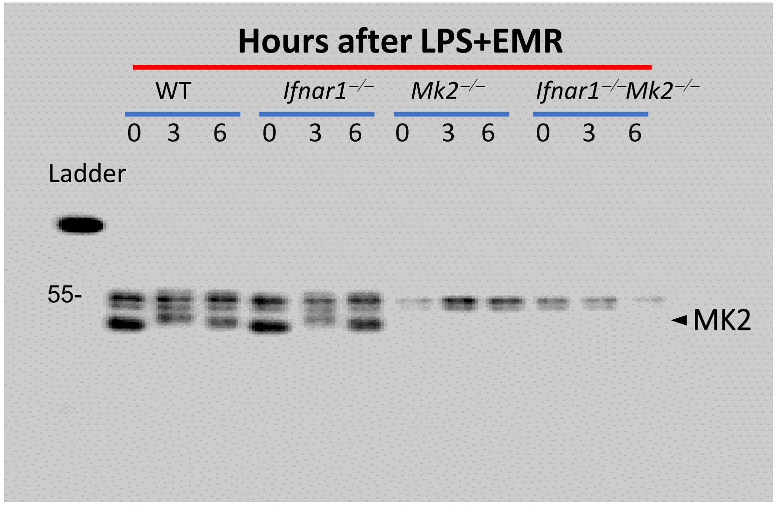

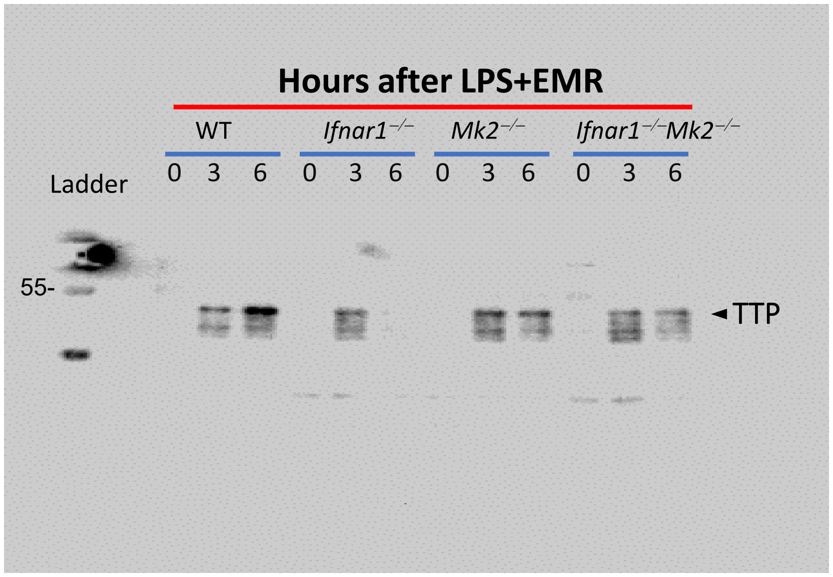

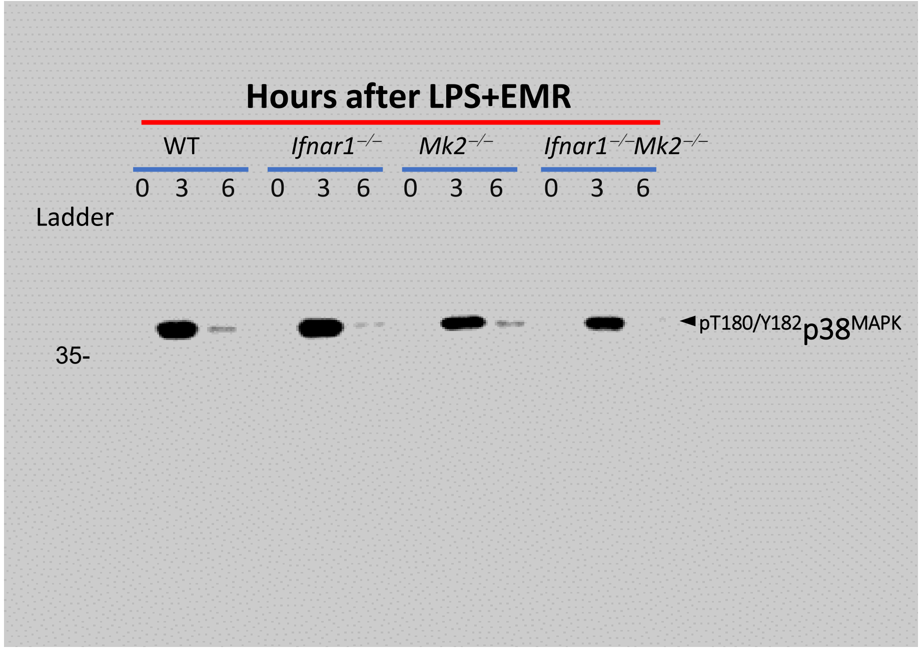

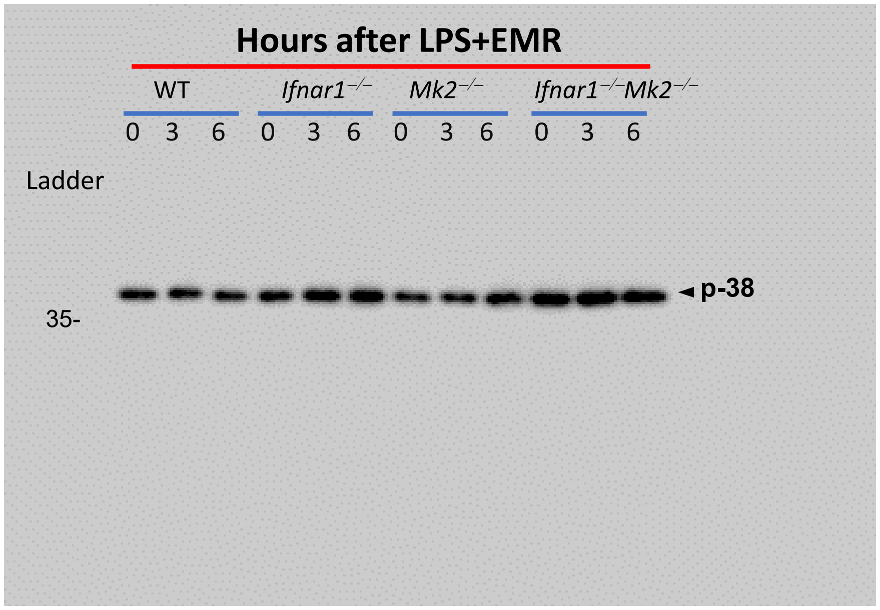

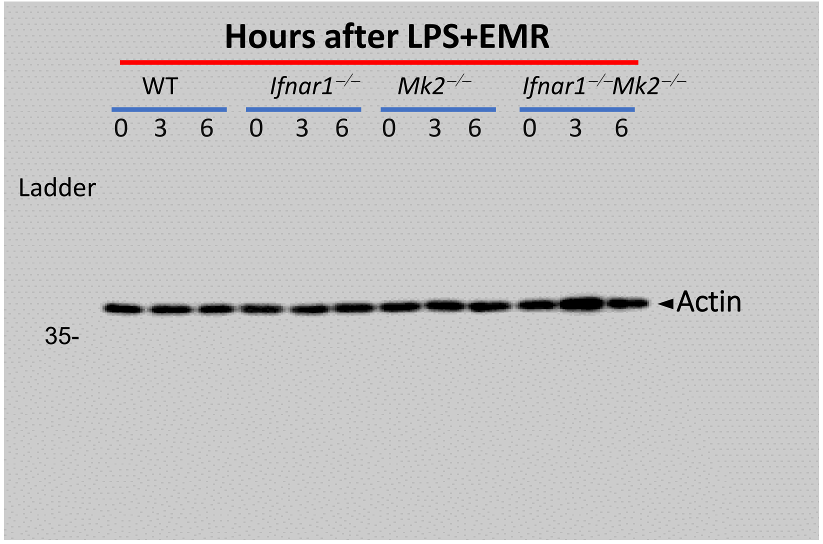

Fig. S1M panels

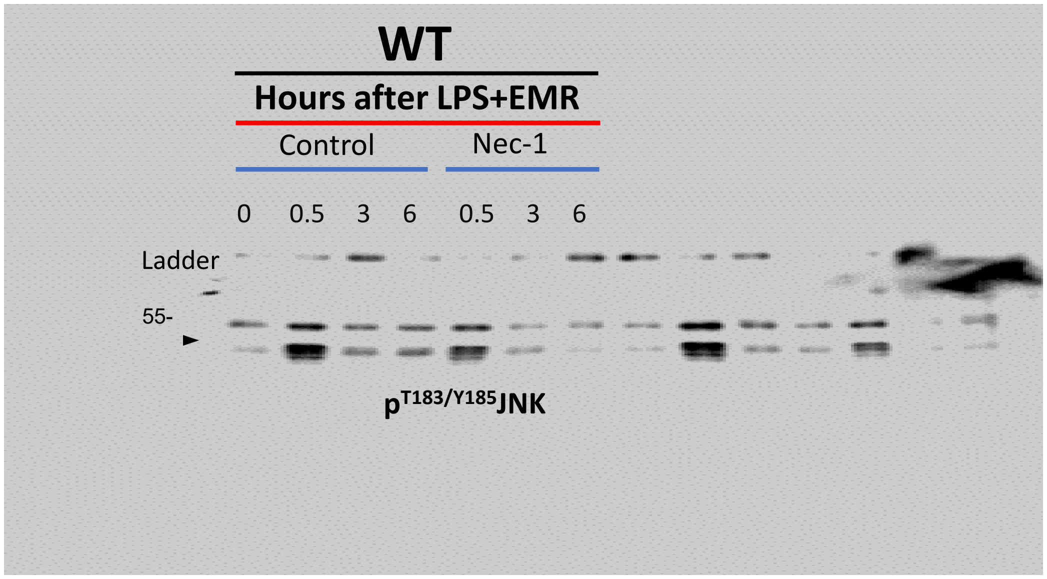

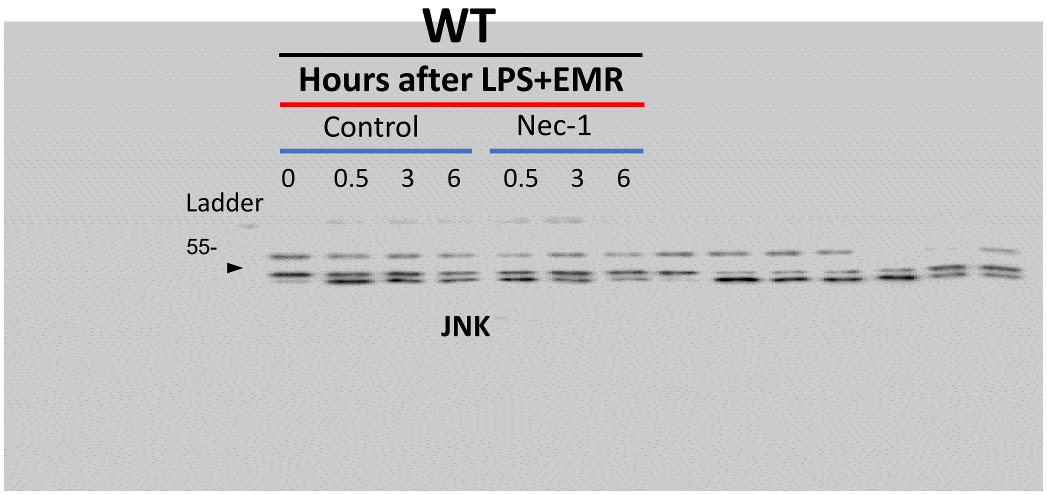

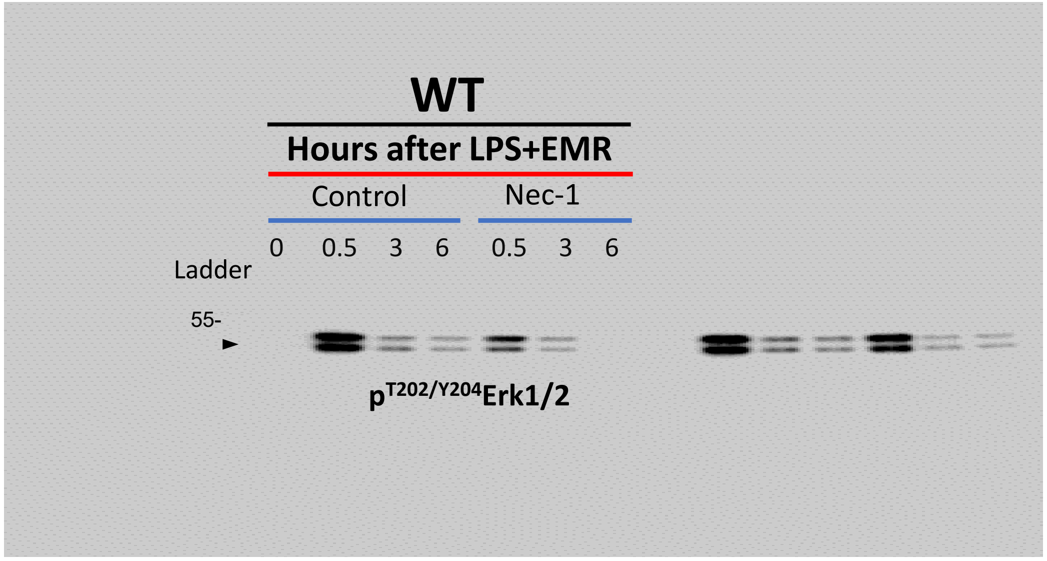

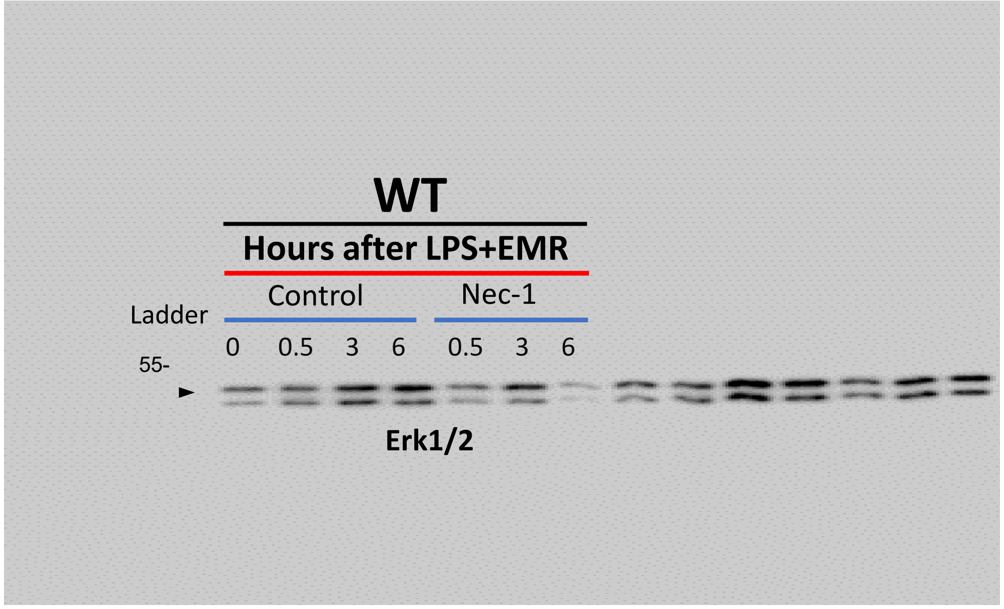

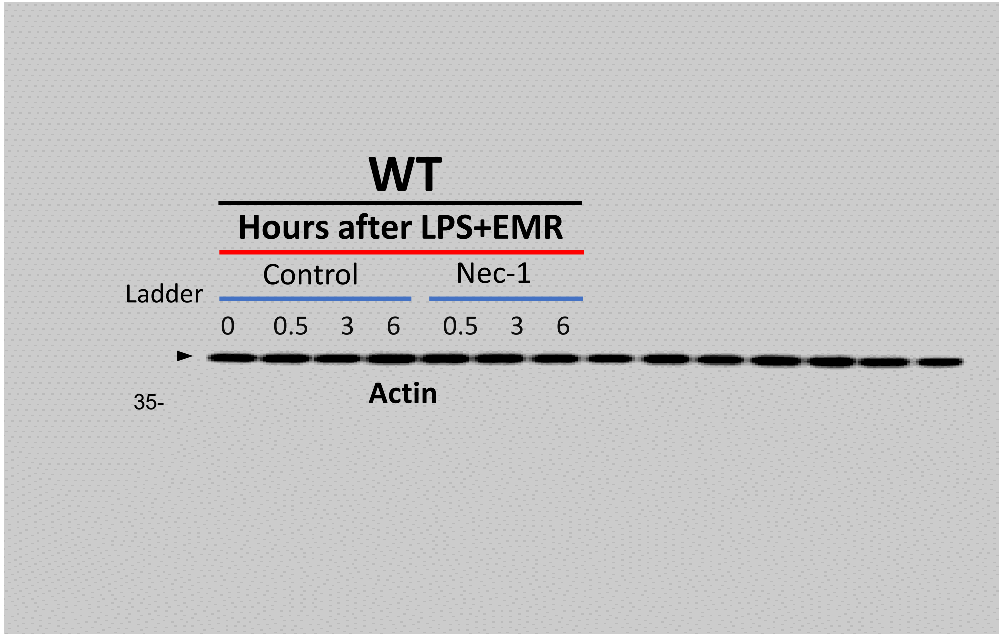

Fig. S3C panels

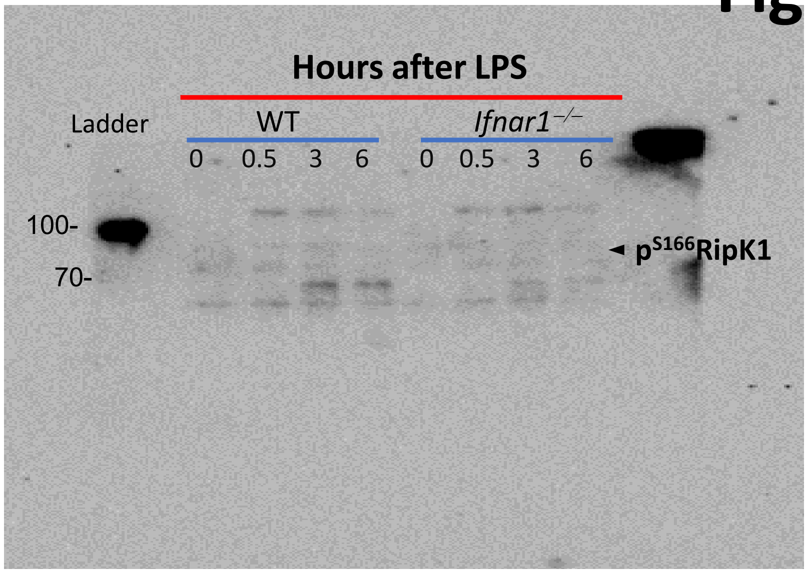

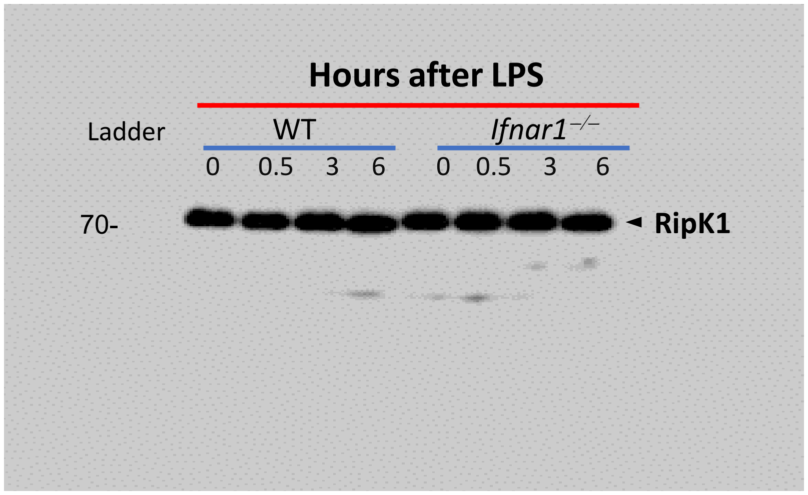

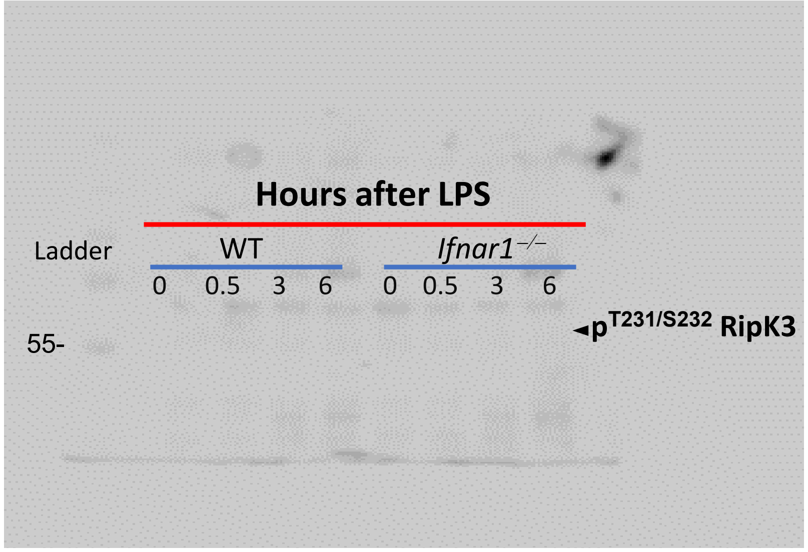

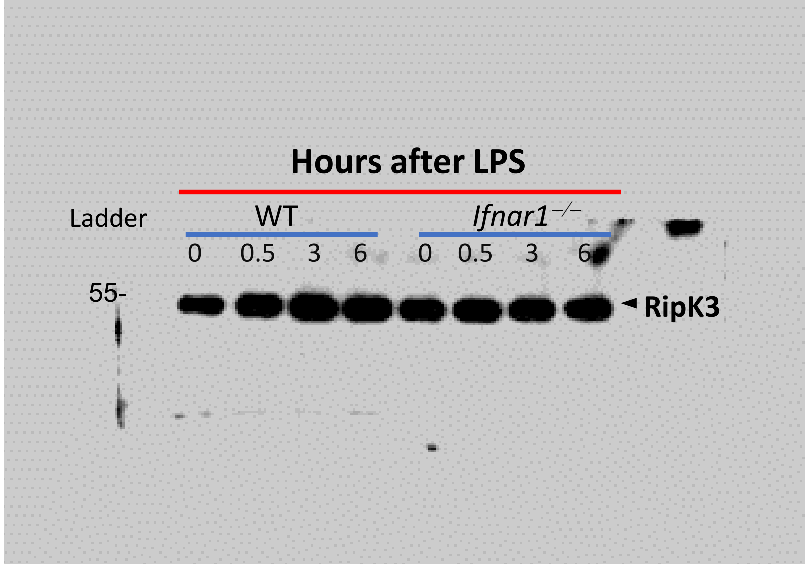

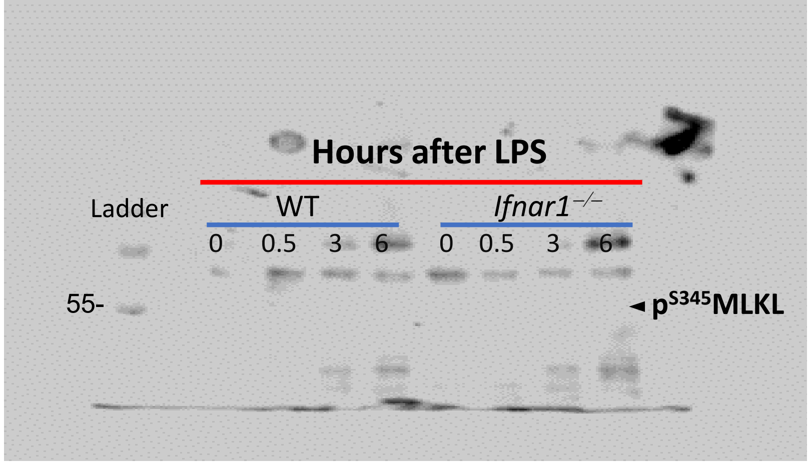

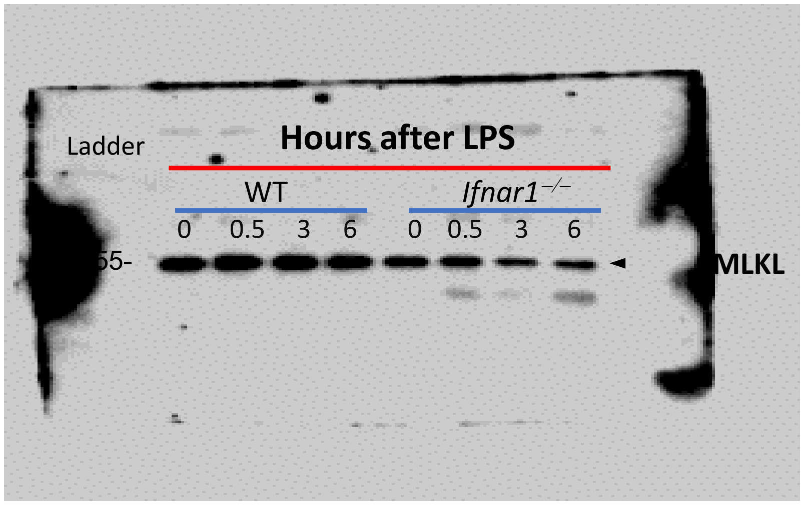

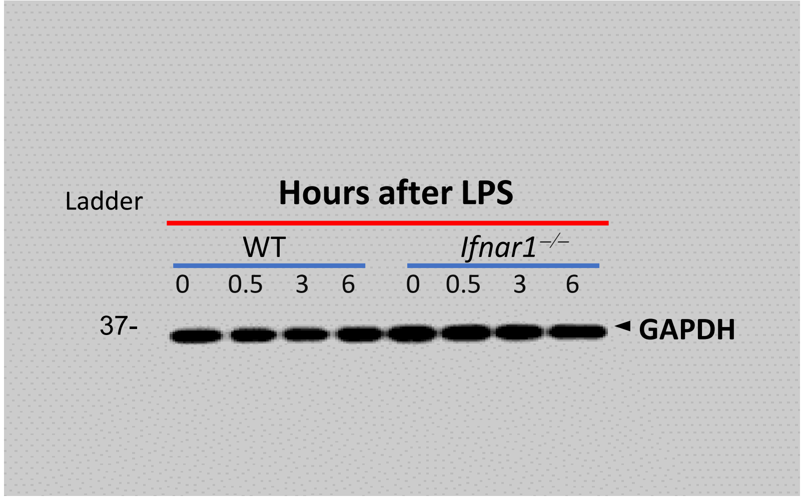

Fig. S3D panels

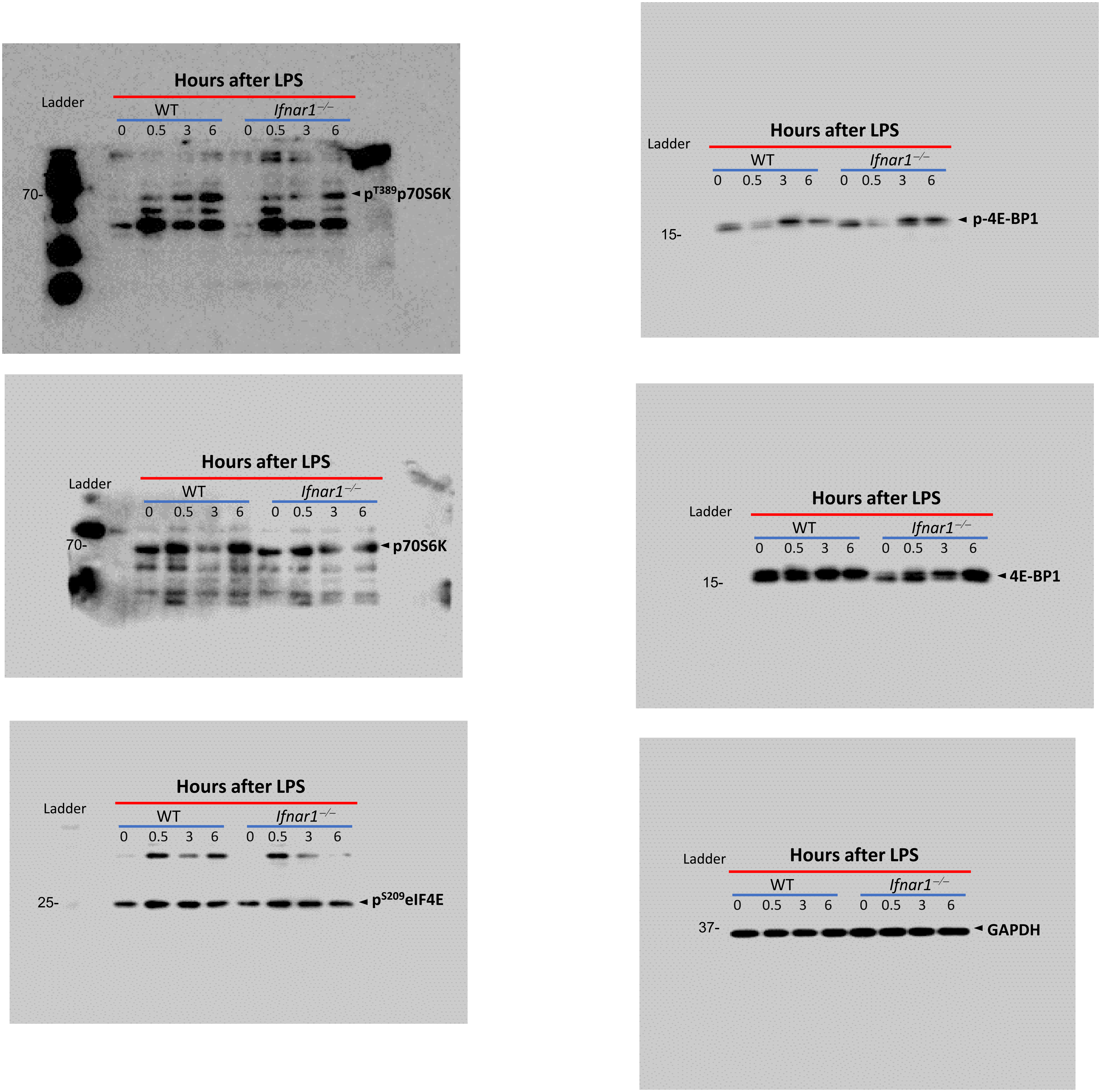

Fig. S5 panels

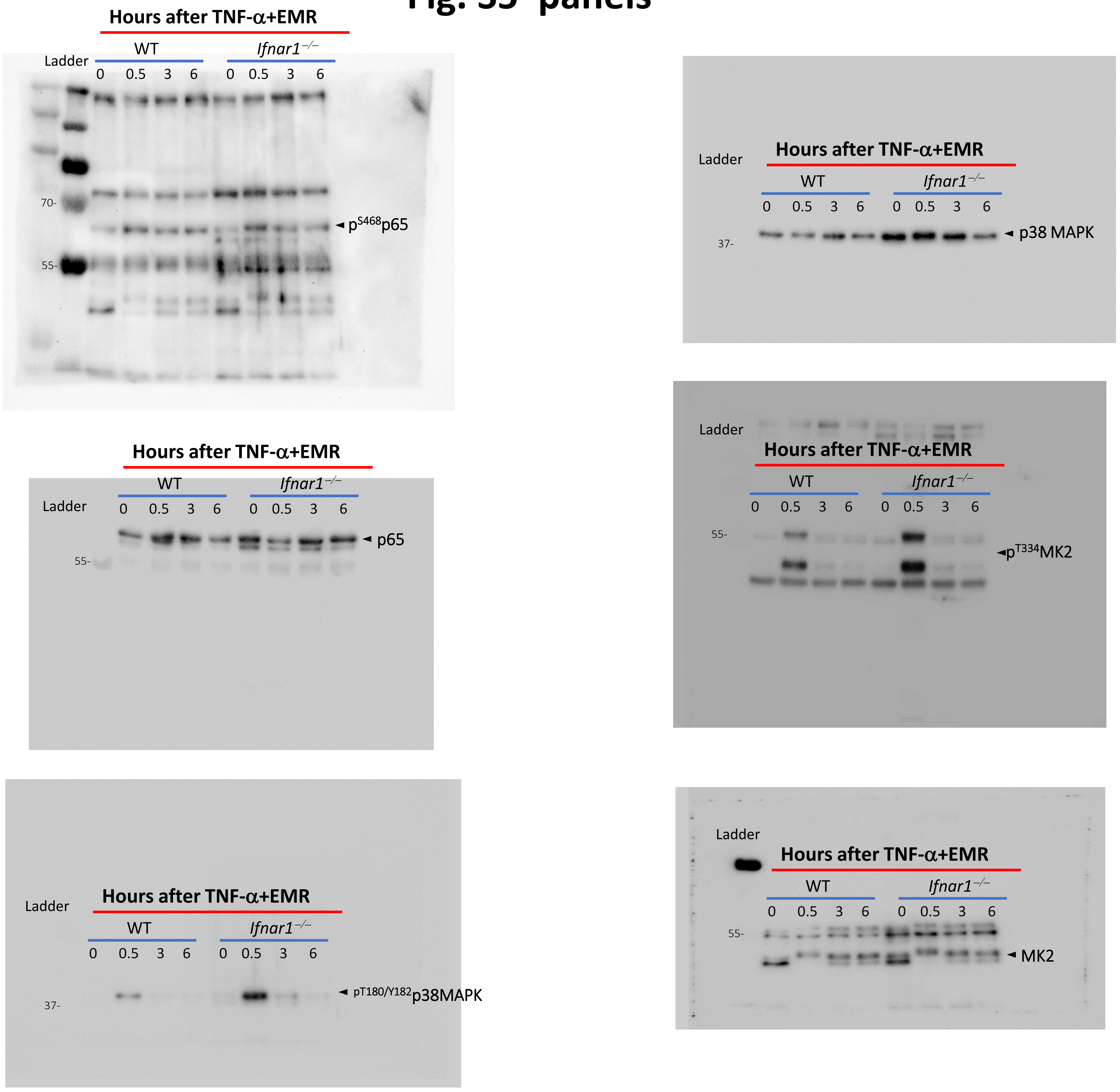

Fig. S5 panels

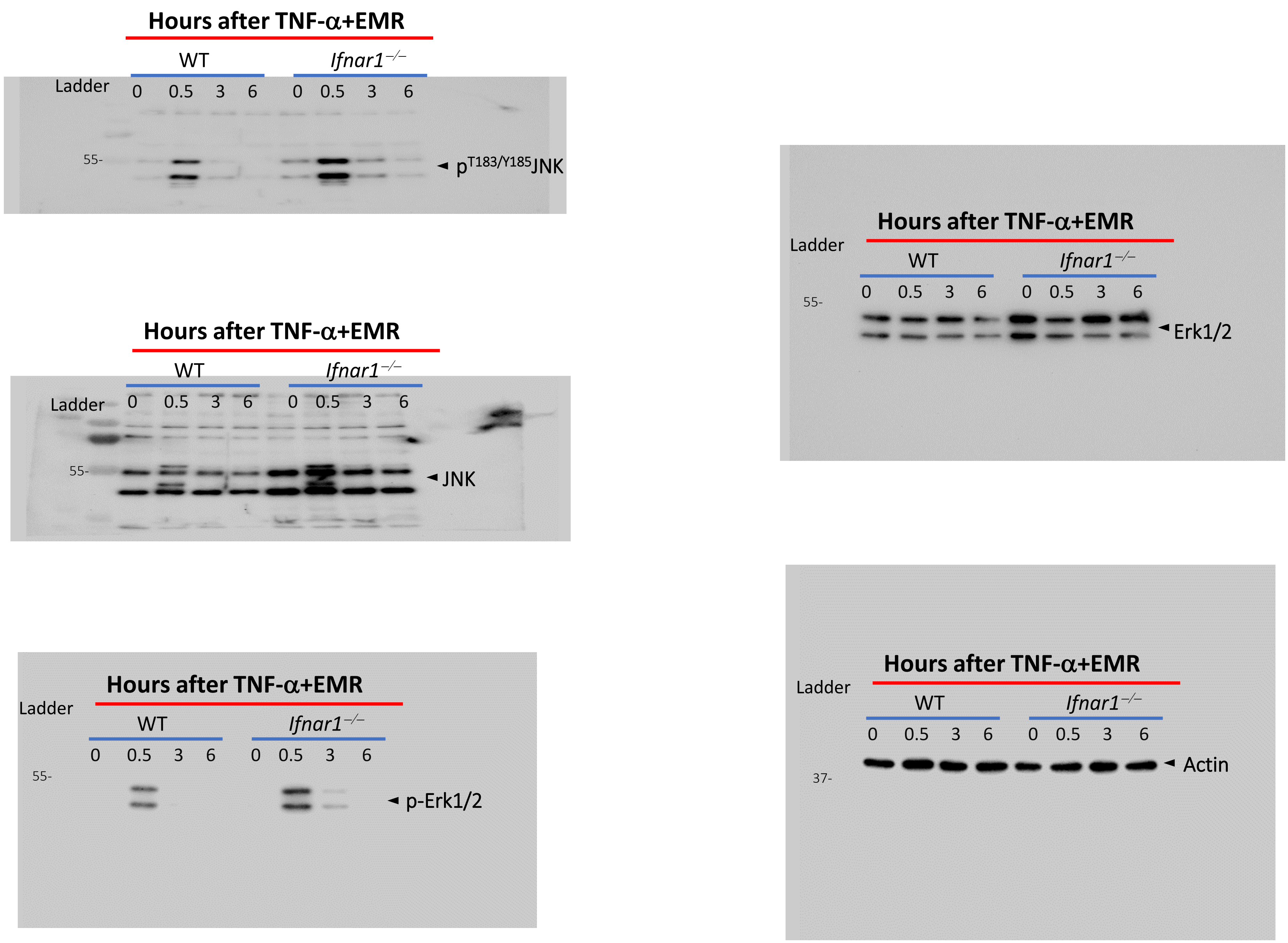

Supplement: Supplementary file 8 — Supplementary material [file 41419_2024_6964_MOESM8_ESM.pdf]
